# Prevention of Hepatitis A Virus Infection in the United States: Recommendations of the Advisory Committee on Immunization Practices, 2020

**DOI:** 10.15585/mmwr.rr6905a1

**Published:** 2020-07-03

**Authors:** Noele P. Nelson, Mark K. Weng, Megan G. Hofmeister, Kelly L. Moore, Mona Doshani, Saleem Kamili, Alaya Koneru, Penina Haber, Liesl Hagan, José R. Romero, Sarah Schillie, Aaron M. Harris

**Affiliations:** ^1^Division of Viral Hepatitis, National Center for HIV/AIDS, Viral Hepatitis, STD, and TB Prevention, CDC; ^2^Department of Health Policy, Vanderbilt School of Medicine, Nashville, Tennessee; ^3^Division of Healthcare Quality Promotion, National Center for Emerging and Zoonotic Infectious Diseases, CDC; ^4^Section of Pediatric Infectious Diseases, Department of Pediatrics, University of Arkansas for Medical Sciences, Little Rock, Arkansas

## Abstract

Hepatitis A is a vaccine-preventable, communicable disease of the liver caused by the hepatitis A virus (HAV). The infection is transmitted via the fecal-oral route, usually from direct person-to-person contact or consumption of contaminated food or water. Hepatitis A is an acute, self-limited disease that does not result in chronic infection. HAV antibodies (immunoglobulin G [IgG] anti-HAV) produced in response to HAV infection persist for life and protect against reinfection; IgG anti-HAV produced after vaccination confer long-term immunity. This report supplants and summarizes previously published recommendations from the Advisory Committee on Immunization Practices (ACIP) regarding the prevention of HAV infection in the United States. ACIP recommends routine vaccination of children aged 12–23 months and catch-up vaccination for children and adolescents aged 2–18 years who have not previously received hepatitis A (HepA) vaccine at any age. ACIP recommends HepA vaccination for adults at risk for HAV infection or severe disease from HAV infection and for adults requesting protection against HAV without acknowledgment of a risk factor. These recommendations also provide guidance for vaccination before travel, for postexposure prophylaxis, in settings providing services to adults, and during outbreaks

## Introduction

The hepatitis A virus (HAV) is transmitted via the fecal-oral route, usually through direct person-to-person contact or consumption of contaminated food or water (*1*,*2*). HAV infection is clinically indistinguishable from other types of acute viral hepatitis, and the illness is usually mild and self-limited when healthy persons are infected (*1*,*2*). Disease severity increases in persons who are older or immunocompromised, have chronic liver disease, or have other underlying health conditions (*2*–*4*). Infection with HAV has not been found to cause chronic infection, although prolonged or relapsing hepatitis A has been reported (*5*).

Recommendations for hepatitis A (HepA) vaccine were introduced incrementally in the United States. In 1996, the Advisory Committee on Immunization Practices (ACIP) recommended routine vaccination of children aged ≥2 years who lived in communities with high rates of HAV infection, for populations at increased risk for HAV infection or the adverse consequences of infection, and in outbreak settings (*6*). In 1999, ACIP expanded the recommendations to include routine vaccination for the following groups: 1) children aged ≥2 years in 11 states (Alaska, Arizona, California, Idaho, Nevada, New Mexico, Oklahoma, Oregon, South Dakota, Utah, and Washington) with average incidence rates that were at least twice the national average during 1987–1997 (i.e., ≥20 cases per 100,000 population) and 2) consideration of routine vaccination of children aged ≥2 years in six states (Arkansas, Colorado, Missouri, Montana, Texas, and Wyoming) where average incidence rates were greater than, but less than twice, the national average (i.e., ≥10 but <20 cases per 100,000 population) (*7*). In 2006, ACIP recommended routine HepA vaccination of all children aged 12–23 months (*8*). These recommendations resulted in a 95.5% decrease in reported hepatitis A cases during 1996–2011 (*9*). Small increases in cases occurred in 2013 and 2016 attributed to foodborne outbreaks associated with contaminated food (*10*,*11*). Beginning in 2016, greater increases in the number of reported cases occurred across the United States, primarily from widespread outbreaks of hepatitis A from person-to-person transmission (*12*). Low adult HepA vaccination coverage and high population susceptibility to HAV infection allow outbreaks to continue to occur (*13*,*14*). This report supplants and summarizes previously published recommendations from ACIP for the prevention of HAV infection in the United States. The recommendations can be used by health care providers to update the current practice for providing HepA vaccines for preexposure and postexposure prophylaxis.

## New or Updated Recommendations

The following ACIP recommendations are new, updated, or no longer recommended:

- Vaccination of all children and adolescents aged 2–18 years who have not previously received HepA vaccine (i.e., children and adolescents are recommended for catch-up vaccination)
- Vaccination of all persons aged ≥1 year infected with human immunodeficiency virus (HIV)
- Vaccination of persons with chronic liver disease, including but not limited to persons with hepatitis B virus (HBV) infection, hepatitis C virus (HCV) infection, cirrhosis, fatty liver disease, alcoholic liver disease, autoimmune hepatitis, or an alanine aminotransferase (ALT) or aspartate aminotransferase (AST) level persistently greater than twice the upper limit of normal
- Vaccination of pregnant women who are identified to be at risk for HAV infection during pregnancy (e.g., international travelers, persons who use injection or noninjection drugs, persons who have occupational risk for infection, persons who anticipate close personal contact with an international adoptee, or persons experiencing homelessness) or for having a severe outcome from HAV infection (e.g., persons with chronic liver disease or persons with HIV infection)
- Vaccination during hepatitis A outbreaks of persons aged ≥1 year who are at risk for HAV infection (e.g., persons who use injection or noninjection drugs [i.e., all those who use illegal drugs], persons experiencing homelessness, or MSM) or who are at risk for severe disease from HAV (e.g., persons with chronic liver disease or who are infected with HIV)
- Vaccination in settings providing services to adults in which a high proportion of persons have risk factors for HAV infection (e.g., health care settings with a focus on those who use injection or noninjection drugs [i.e., all those who use illegal drugs], group homes, and nonresidential day care facilities for developmentally disabled persons)
- Vaccination of persons who receive blood products for clotting disorders (e.g., hemophilia) is no longer recommended.

New CDC clinical guidance is provided for the vaccination of the following: infants aged 6–11 months traveling outside the United States, persons aged >40 years, persons with immunocompromising conditions, and persons with chronic liver disease planning on traveling, persons with HIV infection, pregnant women, postexposure prophylaxis and vaccination during outbreaks.

## Methods

ACIP’s Hepatitis Vaccines Work Group comprises professionals from academic medicine (pediatrics, family medicine, internal medicine, infectious diseases, occupational health, and preventive medicine), federal and state public health agencies, and medical societies. The work group considered previously published ACIP HepA vaccine recommendations, reviewed the epidemiology of and literature for hepatitis A, directed an economic analysis, and deliberated over the recommendations.

This report supplants and summarizes previously published ACIP recommendations for HepA vaccination of children and adults (*8*,*15*–*17*). Data used for general clarifications to the recommendations were summarized based on findings from literature searches that were completed on March 19, 2019, and updated before publication. The literature searches included clinical trials and comparative studies conducted worldwide and published in English since 2005. Epidemiologic and vaccination coverage data were reviewed. All studies yielding pertinent information were deemed relevant for inclusion. The work group organized, evaluated, and discussed information to create the recommendations using the Evidence to Recommendation (EtR) Framework (https://www.cdc.gov/vaccines/acip/recs/grade/etr.html) and Grading of Recommendations Assessment, Development, and Evaluation (GRADE) (https://www.cdc.gov/vaccines/acip/recs/grade/table-refs.html).

The EtR framework was used to review and evaluate data on HepA catch-up vaccination for children and adolescents aged 2–18 years. GRADE was not used to evaluate the evidence for HepA catch-up for several reasons: 1) HepA vaccine has been recommended for administration to children since 1996, 2) HepA vaccine has been recommended for catch-up vaccination based on shared clinical decision-making since 2006, and 3) the efficacy and safety of HepA vaccines has been evaluated and well-documented since 1996 (see Vaccine Safety). The EtR framework tables for HepA catch-up vaccination are available on the ACIP website (https://www.cdc.gov/vaccines/acip/recs/grade/hep-a-catchup-etr.html).

GRADE was performed and the EtR framework was used to review and evaluate data on persons with HIV infection as a risk group for HepA vaccination, and the findings were presented to ACIP at the February and June 2019 meetings. Details on the methods used for GRADE, including the search protocol, databases searched, inclusion criteria, a summary of the evidence, the grading of the evidence, and information on the additional factors considered are provided on the ACIP website (https://www.cdc.gov/vaccines/acip/recs/grade/hep-a-hiv.html), as are the EtR framework tables (https://www.cdc.gov/vaccines/acip/recs/grade/hep-a-hiv-etr.html).

To assess vaccine safety, two post-licensure surveillance systems were searched for adverse events during 2005–2018. The Vaccine Adverse Events Reporting System (VAERS) (https://www.vaers.hhs.gov) is a national passive surveillance system, whereas the Vaccine Safety Datalink (https://www.cdc.gov/vaccinesafety/ensuringsafety/monitoring/vsd) contains population-based vaccine safety studies. Vaccine safety hypotheses can be generated, but not assessed, through VAERS and are subject to limitations including reporting biases and inconsistent data quality (*18*,*19*). The Vaccine Safety Datalink can be used to assess hypotheses that arise from reviews of medical literature, reports to VAERS, changes in vaccination schedules, or the introduction of new vaccines (*20*) (see Vaccine Safety).

During May 2014–June 2019, the work group held 31 teleconferences focused on HepA vaccine topics. Work group and ACIP members also reviewed and commented on a draft of the statement in its entirety before ACIP’s June 2019 meeting. A summary of work group discussions was presented to ACIP on June 27, 2019. At that time, ACIP members voted to approve a draft of the HepA vaccine recommendations, including the recommendation that all children and adolescents aged 2–18 years who have not previously received HepA vaccine be vaccinated (i.e., children and adolescents are recommended for catch-up vaccination) and that all persons with HIV infection aged ≥1 year be vaccinated with HepA vaccine. The ACIP statement was modified during the subsequent review process at CDC to update and clarify wording in the report; however, substantive changes were made only to the clinical guidance on revaccination for persons with HIV, based on ACIP committee deliberations during the June 2019 meeting.

## HAV Background

### Hepatitis A Vaccine Coverage

Among children born during 2015–2016, coverage with ≥2 doses of the HepA vaccine by age 35 months was 76.6% and with ≥1 dose by age 24 months was 84.7% (*21*). In 2017, coverage with ≥2 doses among adolescents aged 13–17 years was 68.4% and with 1 dose was 77.2% (CDC, unpublished data, 2019). In 2017, coverage with ≥2 doses among adults aged ≥19 years was 10.9% overall, with 17.7% coverage among travelers and 20.8% coverage among persons with chronic liver disease (*14*).

### Epidemiology

**FIGURE 1:**
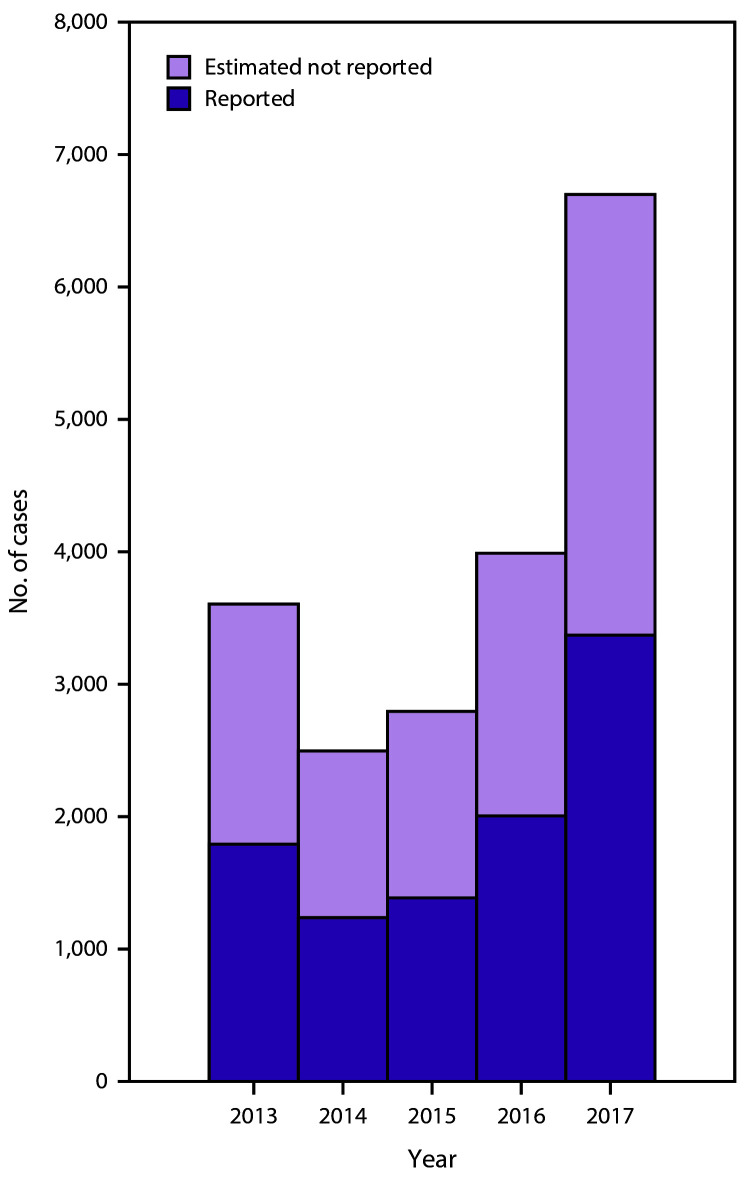
Number of reported and estimated hepatitis A cases — United States, 2013–2017 **Source:** CDC, National Notifiable Diseases Surveillance System. Surveillance for viral hepatitis—United States, 2017. Atlanta, GA: CDC, US Department of Health and Human Services. https://www.cdc.gov/hepatitis/statistics/2017surveillance/index.htm

Preliminary data indicate that in 2018, approximately 12,500 cases of hepatitis A were reported to CDC from 50 states and the District of Columbia (CDC, unpublished data, 2020), representing a substantial increase from 2017 due to widespread outbreaks of hepatitis A from person-to-person transmission (*12*). In 2017, a total of 3,366 cases of hepatitis A were reported to CDC (Figure 1) (*9*). The overall incidence rate of reported cases in 2017 was one case per 100,000 population (*9*). After adjusting for under-ascertainment and underreporting, an estimated 6,700 hepatitis A cases (95% confidence interval [CI]: 4,700–7,400) occurred in 2017 (*9*).

Rates of reported hepatitis A reached a nadir in 2014 for all age groups except those aged 10–19 years, for whom the nadir occurred in 2015. Rates increased in 2016 and 2017 among adults as a result of large community hepatitis A outbreaks with person-to-person transmission among persons who used drugs and persons experiencing homelessness (*9*,*12*,*22*). HAV infections during 2016–2017 also increased among men who have sex with men (MSM) (*23*) and, to a much lesser extent, in association with consumption of imported HAV-contaminated food (*11*).

In 2017, adults aged 30–39 years had the highest hepatitis A rate of all age groups (2.1 cases per 100,000 population), and children aged ≤9 years had the lowest rate (0.1 cases per 100,000 population) (*9*). Rates of HAV infection remain low in children and adolescents because of routine childhood HepA vaccination (*9*). However, rates of HAV infection were higher among adults compared with children and adolescents because of low adult vaccine coverage (*14*) and higher HAV susceptibility (*13*). Among U.S.-born adults aged ≥20 years, HAV susceptibility prevalence (total antibody to HAV negative) was 74.1% (95% CI: 72.9%–75.3%) during 2007–2016 (*13*).

The reported rates of HAV infection among males and females have been similar since 2003 when the rates were 2.82 cases per 100,000 population for males and 2.43 for females (*9*). The rates decreased over time consistent with the national trend and remained similar; however, from 2016 to 2017, rates increased more markedly for males (0.7 cases per 100,000 population in 2016 to 1.38 in 2017) than for females (0.55 cases per 100,000 population in 2016 to 0.7 cases in 2017), consistent with higher rates of drug use among men compared with women (*9*,*24*). In 2017, the rates of reported hepatitis A cases were similar among all racial/ethnic populations. The highest rate was among white, non-Hispanics (0.98 per 100,000 population), and the lowest was among American Indians/Alaska Natives (0.48 per 100,000 population) (*9*).

### Virus Description

HAV has a single-stranded, positive-sense RNA genome that is approximately 7.5 kb long. The virus is a member of the *Picornaviridae* family in the *Hepatovirus* genus (*25*–*28*). Two forms of infectious virions exist (*29*). A naked, nonenveloped virion 27 nm in diameter is shed in the feces of infected persons, and a quasi-enveloped virion is found in the blood of infected persons and secreted nonlytically from infected cells (*29*).

HAV is classified into six genotypes. Genotypes I, II, and III circulate among humans, whereas genotypes IV, V, and VI infect simians (*30*,*31*). The antibody response to HepA vaccines is similar across genotypes (*32*). Only one human HAV serotype has been identified globally (*30*).

### Transmission

HAV is transmitted via the fecal-oral route, usually from direct person-to-person contact or consumption of contaminated food or water (*33*). Children were a key source of HAV transmission before HepA vaccination was available and recommended routinely for children because the majority of children infected with HAV have asymptomatic or unrecognized infections and can shed the virus in their feces for months (*34*,*35*). Transmission currently occurs primarily among susceptible adults.

Common-source outbreaks and sporadic cases also occur from exposure to food or water with fecal contamination. Uncooked foods have been recognized as a source of outbreaks (*36*). Cooked foods also can transmit HAV if the heat level used in preparation is inadequate to inactivate the virus or if food is contaminated after cooking, which can occur during outbreaks associated with infected food handlers (*36*). Waterborne outbreaks of hepatitis A are infrequent in developed countries with well-maintained sanitation and water supplies (*37*). Depending on conditions, HAV can be stable in the environment for months (*33*,*38*). HAV also is stable when frozen (*10*,*39*–*41*). Heating foods at temperatures >185°F (>85°C) for 1 minute or disinfecting surfaces with a 1:100 dilution of sodium hypochlorite (i.e., household bleach) in tap water inactivates HAV (*42*).

On rare occasions, HAV has been transmitted by transfusion of blood or blood products collected from donors during the viremic phase of their infection (*43*,*44*). Since 2002, nucleic acid amplification tests (NAATs) for the detection of HAV RNA have been applied to the screening of source plasma used for the manufacture of plasma-derived products, drastically reducing the transmission risk from plasma-derived products (*45*). 

In experimentally infected nonhuman primates, HAV has been detected in saliva during the incubation period (*46*), as well as in serum and stool samples. HAV also has been detected in human saliva (*47*,*48*); however, no studies assessing the transmission of HAV infection by saliva are available.

### Clinical Features and Natural History

Infection in humans occurs after an average incubation period of 28 days (range: 15–50 days) (*49*,*50*). Symptomatic HAV infection is clinically indistinguishable from other types of acute viral hepatitis but is usually mild and self-limited, with an abrupt onset that can include fever, malaise, anorexia, nausea, and abdominal discomfort, followed within a few days to a week by dark urine, pale stools, and jaundice (*1*). The likelihood of having symptoms with HAV infection is related to age. In children aged <6 years, 70% of infections are asymptomatic; illnesses that do occur are not typically accompanied by jaundice (*1*,*51*). Among older children and adults, infection typically is symptomatic, with jaundice occurring in >70% of patients (*1*,*52*). Signs and symptoms usually resolve within 2–3 months, with complete recovery within 6 months (*53*). However, 10%–15% of symptomatic persons have prolonged or relapsing hepatitis A lasting up to 6 months and should be considered infectious (*5*). Although relapse consists of elevated liver enzyme levels and detection of virus in stools, recovery from relapse is universal (*52*–*54*), and infection with HAV has not been found to cause chronic infection. Fulminant hepatic failure is rare and occurs in <1% of cases (*2*), although hepatic failure has been observed in a higher percentage of cases in the widespread outbreaks that began in 2016 (*12*,*55*).

In infected persons, HAV replicates in the liver, is excreted in bile, and is shed in stool. Peak transmissibility in infected persons occurs during the 2-week period before the onset of jaundice or elevation of liver enzymes, when concentration of virus in stool is highest (*33*). The concentration of virus in the stool decreases after jaundice appears, and most persons cease to be infectious 1 week after jaundice onset (*33*). Children can shed HAV for longer periods than adults, up to 10 weeks (*56*) after onset of clinical illness. In one nosocomial outbreak, infants who were infected as neonates shed HAV for up to 6 months (*57*). Chronic shedding of HAV in feces does not occur typically; however, recurrent shedding occurs during relapses in persons with relapsing illness (*54*). Viremia occurs soon after infection and persists through the period of liver enzyme elevation but at concentrations several orders of magnitude lower than in stool (*43*,*56*–*58*). Prolonged viremia and fecal shedding of HAV for more than a month in immunocompetent persons and for more than a year in immunocompromised patients has been reported in several studies (*59*,*60*). Prolonged viremia after organ transplantation also has been described (*61*).

### Interpretation of Serologic Markers and Diagnosis of HAV Infection

**FIGURE 2:**
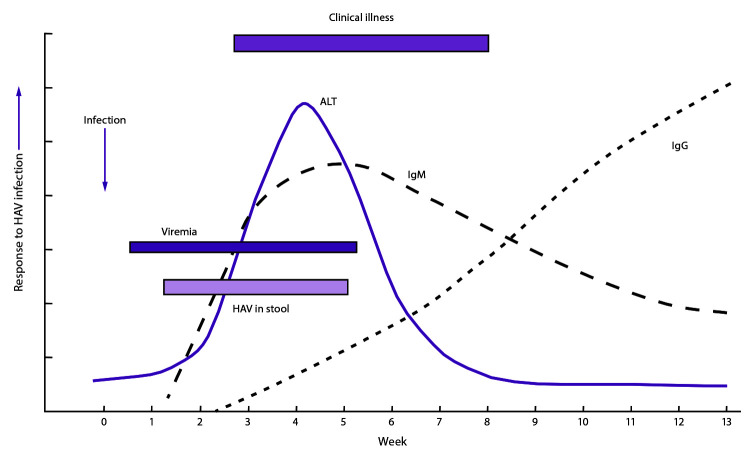
Immunologic and clinical events associated with hepatitis A virus infection and recovery **Source:** CDC. Diagnosis and management of foodborne illnesses. MMWR Recomm Rep 2004;53(No. RR-4). **Abbreviations:** ALT = alanine aminotransferase; HAV = hepatitis A virus; IgG = immunoglobulin G; IgM = immunoglobulin M.

Hepatitis A cannot be differentiated from other types of viral hepatitis on the basis of clinical or epidemiologic features alone. Diagnosis of HAV infection requires the detection of either immunoglobulin M (IgM) anti-HAV in serum or HAV RNA in serum or stool. IgM anti-HAV, a marker of acute illness, becomes detectable within 5–10 days of the onset of symptoms in the majority of persons (*58*,*62*), usually peaks within 1 month of illness, and decreases to undetectable levels within 6 months after infection (*58*,*62*–*64*) (Figure 2). However, positive tests for IgM anti-HAV in persons >1 year after infection have been reported, as have likely false-positive tests in persons without evidence of recent HAV infection (*65*–*67*). Therefore, to reduce false-positive tests, persons should only be tested for IgM anti-HAV if they are symptomatic and suspected of having HAV infection (*66*–*68*). Total anti-HAV or immunoglobulin G (IgG) anti-HAV testing is used in epidemiologic studies to measure the prevalence of previous infection or clinically to determine whether a person with an indication for preexposure prophylaxis is already immune. IgG anti-HAV is produced either in response to natural infection or after vaccination with HepA vaccine, appearing early in the course of infection, remaining detectable for the person’s lifetime, and providing lifelong protection against the disease (*69*–*71*). The antibody test for total anti-HAV measures both IgG anti-HAV and IgM anti-HAV. Although assays for the detection of IgG anti-HAV are available, in the absence of an IgG anti-HAV test, a total anti-HAV–positive sample from a symptomatic person should be subsequently tested for IgM anti-HAV to establish the acute stage of HAV infection (*72*). Persons who test positive from a total anti-HAV test and negative from an IgM anti-HAV test are considered immune, either from past infection or vaccination.

Biochemical evidence of hepatitis includes elevated levels of serum bilirubin and serum hepatic enzymes, including ALT, AST, alkaline phosphatase, and gamma-glutamyltranspeptidase. Elevations in AST and ALT levels usually occur 5–10 days before symptom onset. Serum bilirubin and aminotransferase levels usually return to normal by 2–3 months after illness onset (*53*).

Molecular epidemiologic technologies, when applied in combination with conventional epidemiologic methods, have been useful in investigating outbreaks and determining transmission links (*32*). The detection and sequencing of HAV RNA from water, food, and blood or stool of infected persons provide important information for tracing and characterizing HAV strains and identifying the source of water and foodborne hepatitis A outbreaks. HAV RNA can be detected using NAAT technologies in the blood and stool of the majority of persons during the acute phase of infection using NAAT technologies (*73*–*75*). HAV RNA in liver biopsies, cell cultures, or environmental samples can also be detected using various NAAT-based research methodologies (*76*). Growth of HAV in cell culture requires a long adaptation period and is associated with acquisition of various adaptive mutations in the viral genome (*77*,*78*). However, detection of serologic markers is sufficient for routine surveillance.

### Adults at Risk for HAV Infection or for Severe Disease from HAV

#### International Travelers

**FIGURE 3:**
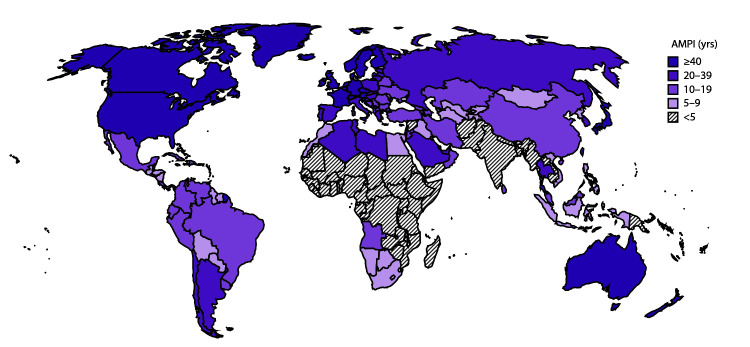
Estimated age at midpoint of population immunity* **to hepatitis A, by country — 2015** **Source:** Jacobsen KH. Globalization and the changing epidemiology of hepatitis A virus. Cold Spring Harb Perspect Med 2018;8:a031716. **Abbreviations:** AMPI = age at midpoint of population immunity; HAV = hepatitis A virus. * The AMPI is the youngest age at which half of the birth cohort has serologic evidence of previous exposure to HAV. As the AMPI increases (light to dark), the endemicity level of hepatitis A generally decreases.

Unvaccinated persons from developed countries who travel to countries that have high or intermediate hepatitis A endemicity have a substantial risk for acquiring hepatitis A (*9*,*79*). Endemicity is related to the age at midpoint of population immunity (AMPI); as the AMPI increases, the endemicity level of hepatitis A generally decreases (*80*) (Figure 3). However, determining global HAV endemicity is complex, and limited data are available on subpopulation variation of anti-HAV seroprevalence within regions (*80*,*81*). Travelers at risk include tourists, nonimmune immigrants and their children returning to their country of origin to visit friends or relatives, military personnel, missionaries, and others who work or study abroad. Hepatitis A remains one of the most common vaccine-preventable diseases acquired during travel (*82*). Risk is highest for those who live in or visit rural areas, trek in backcountry areas, or frequently eat or drink in settings with poor sanitation. However, cases of travel-related hepatitis A can occur in travelers who have tourist itineraries, accommodations, and eating behaviors that are considered low risk. Travelers who acquire hepatitis A during their trips might transmit HAV to others on their return.

#### Men Who Have Sex with Men

Hepatitis A outbreaks among MSM have been reported frequently (*83*,*84*). Since 2016, multiple hepatitis A outbreaks have been reported among MSM linked to travel in areas with ongoing HAV transmission among MSM (e.g., in >20 European Union countries) (*23*,*85*–*91*). Molecular epidemiology shows similar HAV strains circulating among HAV-infected MSM, suggesting transmission chains occur in this population (*74*). ACIP has recommended routine HepA vaccination of MSM since 1996 (*6*). Despite this longstanding recommendation, vaccination coverage among MSM remains suboptimal, with vaccination coverage estimated at 25%–45% overall (*13*,*92*–*94*), although vaccination coverage might be higher in targeted high-risk settings (*95*).

#### Persons Who Use Injection or Noninjection Drugs

Outbreaks occur frequently among persons who use injection or noninjection drugs (i.e., all those who use illegal drugs) (*12*,*55*,*96*–*98*). Transmission among persons who use injection or noninjection drugs occurs via the fecal-oral route (resulting from poor hygiene practices or lack of adequate sanitation) and also might occur via the percutaneous route among persons who inject drugs (*96*,*98*); however, HAV RNA levels are several log_10_ units lower in serum than in feces. Since 1996, ACIP has recommended HepA vaccination for persons who use injection or noninjection drugs (*6*). Despite this longstanding recommendation, HAV seropositivity remains low (20%–40%) in this population (*13*,*99*,*100*).

#### Persons with Occupational Risk for Exposure

Outbreaks of hepatitis A have been reported among persons working with nonhuman primates, including Old World and New World species (*101*,*102*). Primates that were infected had been born in the wild (*103*). Persons working with clinical or nonclinical material containing HAV in a research laboratory setting are considered at risk for HAV infection. 

Health care personnel are not at substantially increased risk for HAV infection through occupational exposure, and health care–associated HAV transmission is rare (see Groups and Settings with Low Risk for Hepatitis A) (*104*). Food handlers are also not considered at increased risk for HAV infection (see Groups and Settings with Low Risk for Hepatitis A)(*36*).

#### Persons Who Anticipate Close Personal Contact with an International Adoptee

HAV infection can occur among family members of international adoptees (e.g., have close personal contact) (*16**,**105*). The majority of children adopted from foreign countries by families in the United States come from countries where hepatitis A is of high or intermediate endemicity, which is related to the AMPI; as the AMPI increases, the endemicity level of hepatitis A generally decreases (Figure 3) (*80*). Approximately 50 cases of HAV infection have been reported after exposure to international adoptees (*16*,*106*–*110*), likely an underestimate because data regarding contact with an international adoptee is not routinely collected as part of national hepatitis A surveillance. The risk for HAV infection among close personal contacts of international adoptees was estimated at 106 per 100,000 household contacts of international adoptees within the first 60 days of their arrival in the United States (CDC, unpublished data, 2009). By comparison, according to surveillance data, the estimated rate of symptomatic hepatitis A in the U.S. general population in 2009 was 0.6 cases per 100,000 population (*111*).

#### Persons Experiencing Homelessness

A homeless person is defined as 1) a person who lacks housing (regardless of whether the person is a member of a family), including a person whose primary residence during the night is a supervised public or private facility (e.g., shelter) that provides temporary living accommodations and a person who is a resident in transitional housing, 2) a person without permanent housing who might live on the streets; or stay in a shelter, mission, single-room occupancy facility, abandoned building, vehicle, or any other unstable or nonpermanent situation, or 3) who is “doubled up,” a term that refers to a situation where persons are unable to maintain their housing situation and are forced to stay with a series of friends or extended family members. In addition, previously homeless persons who are to be released from a prison or a hospital might be considered homeless if they do not have a stable housing situation to which they can return. A recognition of the instability of a person’s living arrangements is critical to the definition of homelessness (*112*–*114*).

In 2017, outbreaks caused by person-to-person HAV transmission among persons experiencing homelessness signaled a shift in hepatitis A epidemiology in the United States (*12*). Persons experiencing homelessness might have difficulty implementing recommended nonvaccine strategies to protect themselves from exposure (e.g., access to clean toilet facilities, regular handwashing, and avoidance of crowded living conditions). For this reason, vaccination is the most reliable protection from HAV infection for persons experiencing homelessness. HepA vaccination of persons experiencing homelessness provides individual protection and increases herd immunity, reducing the risk for large-scale outbreaks from person-to-person transmission in this population (*15*). In addition, homelessness is associated with other known risk behaviors for HAV infection (e.g., drug use) (*115*).

#### Persons with HIV Infection

Data suggest that up to 87% of persons with HIV infection are susceptible (i.e., have negative total or IgG anti-HAV test results) to HAV infection because of lack of previous HAV infection, lack of receipt of HepA vaccination, or poor response to HepA vaccination (*116*–*122*). Persons with HIV infection who have underlying liver disease are at increased risk for severe disease from HAV infection (*123*,*124*). HAV infection in persons with HIV infection is prolonged and might increase HIV replication, potentially increasing HIV transmission, and can lengthen the HAV transmission period (*124*–*132*). Protracted hepatitis A outbreaks can occur among persons with HIV infection (*133*,*134*) because HAV viremia in these persons tends to be elevated and the hepatitis A clinical course tends to be prolonged compared with HIV-uninfected persons (*131*), resulting in a higher risk for HAV transmission (*131*,*133*).

HepA vaccine seroconversion rates (i.e., achieving IgG anti-HAV levels after vaccination that are higher than the correlate of protection as determined by the study) in persons with HIV infection range from 50% to 94% (*135*,*136*) (see Correlate of Protection). HepA vaccine does not increase HIV viral load, affect CD4 cell count, or accelerate progression to acquired immunodeficiency syndrome (AIDS) (*137*–*142*). Protection against HAV in persons with HIV infection can be achieved, despite lower seroconversion rates compared with HIV-negative persons (see Vaccine-Induced Seroprotection) (*136*,*143*,*144*). In one study among 130 persons with HIV infection, 85% maintained seropositivity 6–10 years after a 2-dose vaccine series (*145*). Vaccination at higher CD4 counts is associated with better vaccine-induced immune responses (*135*,*136*,*141*,*145*–*147*).

#### Persons with Chronic Liver Disease

Persons with chronic liver disease are not at increased risk for acquiring HAV infection. However, concurrent underlying chronic liver disease (e.g., HBV infection, HCV infection, cirrhosis, fatty liver disease, alcoholic liver disease, and autoimmune hepatitis) has been associated with increased risk for fulminant hepatitis when HAV infection occurs (*3*,*148*–*152*). Among patients hospitalized for HAV infection in the United States during 2010–2011, the percentage hospitalized with any liver disease (e.g., other viral hepatitis, non-alcohol–related cirrhotic liver disease, alcohol-related liver disease, liver disease not otherwise specified, and biliary tract disease) was high (38.3%; 2.7 per 100,000 population) (*153*).

#### Persons Living in Group Settings for Those with Developmental Disabilities

Historically, HAV infection was highly endemic in institutions for persons with developmental disabilities (*154*). Because fewer children with developmental disabilities have been institutionalized and as conditions in institutions have improved, the incidence and prevalence of HAV infection in these settings have decreased (*8*). Persons with developmental disabilities who require assistance with activities of daily living often live in group homes or small residential facilities. Outbreaks have occurred in these settings and are associated with poor hand hygiene, wearing diapers, and living in close quarters (*155*,*156*).

#### Persons Who Are Incarcerated

Persons with a history of using drugs or homelessness are overrepresented in correctional facilities and often cycle frequently between correctional facilities and the community (*157*,*158*). Ongoing drug use within incarcerated populations, congregate and sometimes overcrowded living conditions, and shared hygiene facilities can increase HAV transmission within jails and prisons (*157*).

Offering HepA vaccine in jails and prisons is an effective strategy to reach persons at high risk for HAV infection who are otherwise difficult to access in the community. Corrections-based vaccination programs have played a role in mitigating communitywide outbreaks of hepatitis A in the United States, the United Kingdom, and Australia (*96*,*159*,*160*). These programs also have been successfully implemented to limit HAV transmission inside jails and prisons (*159*,*160*). Observational data suggest that partnerships with correctional staff and intensive education efforts can lead to >90% uptake of the first dose of a Twinrix (combined HepA and hepatitis B [HepB] vaccine) series within correctional facilities (*161*,*162*). Accelerated dosing schedules also have been used to increase the likelihood that multiple doses of a Twinrix vaccine series are administered before release from short-term incarceration (*163*).

#### Older Adults (Aged >40 Yrs)

HAV infections are usually symptomatic among adults. The severity of hepatitis A disease increases with age (*1*). Adults aged >40 years are more likely to be hospitalized after HAV infection (*153*).

### Groups and Settings with Low Risk for Hepatitis A

#### Persons with Blood Clotting Disorders

Persons who receive blood products for clotting disorders (e.g., hemophilia) are no longer specifically recommended to receive HepA vaccine. Such persons were first recommended to receive HepA vaccination in 1996 (*6*). Previously, some viral inactivation processes focused only on treatment of blood products with solvents and detergents, which inactivated lipid-enveloped viruses (*164*) but not nonenveloped viruses such as HAV, resulting in a risk for infection (*165*). Secondary virus reduction steps are now common and have closed this gap (*166*–*168*). In addition, in the United States, >80% of persons with clotting disorders receive recombinant clotting factor concentrates, which are sterilized (e.g., pasteurization, heat inactivation, and filtration), eliminating the risk for HAV contamination (*168*). The risk for HAV transmission via transfusion of blood products among persons with clotting disorders is now considered the same as that among the general population (*168*), and source plasma is now screened for HAV (*167*).

#### Food Service Establishments and Food Handlers

HepA vaccination is not specifically recommended for persons who handle food in the absence of other risk factors. Foodborne hepatitis A outbreaks occur relatively infrequently in the United States; however, recent outbreaks of hepatitis A related to pomegranate arils (the fruit-coated seeds) imported from Turkey, frozen scallops imported from the Philippines, and frozen strawberries imported from Egypt demonstrated the risk for outbreaks related to foods imported from HAV-endemic areas (*10*,*11*,*169*). Contamination of food with HAV can happen at any point: growing, harvesting, processing, handling, or after cooking. Food handlers are not at increased risk for hepatitis A because of their occupation (*36*). Transmission of HAV from infected food handlers to susceptible consumers or restaurant patrons in the workplace is rare (*36*,*170*,*171*). Transmission among food handlers has not been common since the adoption of the universal childhood HepA vaccination recommendation in 2006, despite costly and resource-intensive investigations of HAV infections among food handlers. One study found that in >90% of case investigations of infected food handlers, only the food handler was infected, with no secondary cases (*172*). A survey of state health departments experiencing person-to-person hepatitis A outbreaks during 2016–2019 demonstrated that among almost 23,000 hepatitis A outbreak cases reported from states, <4% occurred among food handlers; secondary infections among patrons accounted for 0.2% of outbreak cases (*173*). The risk for secondary infection from hepatitis A–infected food handlers to food establishment patrons in these person-to-person hepatitis A outbreaks was <1% (*173*).

#### Child Care Centers

In the past, hepatitis A outbreaks occurred among children attending child care centers and employees of those centers; however, the outbreak frequency decreased following widespread adoption of universal childhood vaccination (*9*,*51*,*174*). Hepatitis A outbreaks in child care centers are now rare. Historically, poor hygiene among children who wore diapers and the staff who handled and changed diapers contributed to the spread of HAV infection in child care centers; outbreaks rarely occurred in child care centers in which care was provided only to children who were toilet trained (*51*,*174*). Because HAV infection among young children is typically mild or asymptomatic, child care center outbreaks were often only identified when adult contacts (typically parents) became ill (*51*,*174*).

#### Schools

HepA vaccination is recommended for all children. Before universal childhood vaccination, the occurrence of hepatitis A cases in U.S. elementary or secondary schools typically reflected disease acquisition in the community, and HAV transmission in school settings was uncommon (and remains uncommon). If multiple cases occur among children at a school, a common source of infection should be investigated (*39*,*75*).

#### Health Care Institutions

HepA vaccine is not routinely recommended for health care personnel. Health care personnel are all paid and unpaid persons serving in health care settings who have the potential for direct or indirect exposure to patients or infectious materials, including body substances (e.g., blood, tissue, and specific body fluids); contaminated medical supplies, devices, and equipment; contaminated environmental surfaces; or contaminated air (*175*). These personnel include but are not limited to emergency medical service personnel, nurses, nursing assistants, physicians, technicians, therapists, phlebotomists, pharmacists, students and trainees, contractual staff not employed by the health care facility, and persons in departments not directly involved in patient care but who could be exposed to infectious agents that can be transmitted in the health care setting (e.g., clerical, dietary, environmental services, laundry, security, engineering and facilities management, administrative, billing, and volunteer) (https://www.cdc.gov/infectioncontrol/pdf/guidelines/infection-control-HCP-H.pdf) (*175*).

Health care–associated HAV transmission is rare. In the past, outbreaks were observed in neonatal intensive care units when infants acquired infections from transfused blood and subsequently transmitted HAV to other infants and staff (*57*,*176*,*177*). Health care personnel are not at substantially increased risk for HAV infection through occupational exposure (*104*). Outbreaks of hepatitis A caused by transmission from adult patients to health care personnel have typically been associated with fecal incontinence and inadequate hand hygiene (*61*,*178*). However, the majority of hospitalized patients who have hepatitis A are admitted after onset of jaundice, when they are beyond the point of peak infectivity (*179*,*180*). Health care personnel should be encouraged to adhere to recommended infection control practices, standard precautions, and contact precautions for incontinent patients, including hand hygiene (*181*).

#### Workers Exposed to Sewage

HepA vaccination is not routinely recommended for persons handling untreated sewage. Data from serologic studies conducted outside the United States indicate that workers who had been exposed to sewage had a possible elevated risk for HAV infection; however, these analyses did not control for other risk factors (e.g., age or travel to endemic areas) (*182*–*184*). Published reports of three serologic surveys conducted among U.S. wastewater workers and appropriate comparison populations did not identify any substantial or consistent increase in the prevalence of anti-HAV among wastewater workers (*185*–*187*). In addition, in the United States, floods, which can carry raw sewage, are unlikely to cause outbreaks of communicable diseases, and outbreaks of HAV caused by flooding have not been reported (*36*,*188*).

#### Public Water Systems

Hepatitis A outbreaks have previously been reported among persons using private or community wells, swimming pools, or public drinking water contaminated by sewage from nearby septic systems (*36*,*37*,*189*–*194*). Water treatment processes and dilution within public water systems render HAV noninfectious (*36*). No hepatitis A outbreaks associated with drinking water have been reported since 2009 in the United States (*37*). Recreational water venues (e.g., spas and swimming pools) that are adequately treated and are not contaminated (e.g., by sewage or children in diapers) are unlikely to pose a risk for hepatitis A outbreaks (*36*,*192*). 

### Outbreaks

Most cases of hepatitis A in the United States result from person-to-person transmission during communitywide outbreaks (*195*,*196*). Since 2016, communitywide outbreaks have occurred predominantly among adults with specific risk factors (e.g., persons who use drugs, persons experiencing homelessness, and MSM) (*12*). However, common-source outbreaks from contaminated food occur as well. Foods that have been implicated in multiple hepatitis A outbreaks (e.g., lettuce, green onions, strawberries, raspberries, and pomegranate arils) have typically been eaten raw (*10*,*39*,*169*,*197*–*200*). For example, a large outbreak (approximately 600 infected) at a single restaurant was associated with imported green onions (*199*,*200*). Shellfish-associated outbreaks have occurred after consumption of raw or partially cooked oysters, clams, scallops, or mussels harvested from contaminated waters (*11*,*201*). Contaminated food outbreaks continue to occur and remain a risk (e.g., a multistate outbreak linked to frozen strawberries and a Hawaii outbreak linked to frozen scallops eaten raw, both occurring in 2016) (*11*,*169*).

## Prophylaxis Against HAV Infection

### Hepatitis A Vaccines

Vaccines containing HAV antigen that are licensed in the United States are the single-antigen vaccines Havrix and Vaqta and the combination vaccine Twinrix (containing both HAV and HBV antigens). All are inactivated vaccines. The three vaccines were licensed by the U.S. Food and Drug Administration (FDA): Havrix in 1995, Vaqta in 1996, and Twinrix in 2001 (*202*).

### Preparation

Inactivated HepA vaccines are prepared using methods similar to those used for inactivated poliovirus vaccine (*203*,*204*). HepA vaccines contain an aluminum adjuvant. Havrix and Twinrix contain 2-phenoxyethanol as a preservative, and Vaqta is formulated without a preservative. None of the HepA vaccines licensed in the United State contain thimerosal as a preservative. For Havrix and Twinrix the final vaccine potency (per dose) is expressed as enzyme-linked immunosorbent assay (ELISA) units of HAV antigen. The antigen content for Vaqta is expressed as units of HAV antigen. The manufacturer package inserts include additional information on HepA vaccine preparation (*202*).

### Vaccine Storage and Shipment

HepA vaccine should be stored and shipped at temperatures ranging from 36°F to 46°F (2°C to 8°C) and should not be frozen (*205*). The manufacturer package inserts include additional information (*202*).

### Route of Administration

The vaccine should be administered intramuscularly into the anterolateral aspect of the thigh or the deltoid muscle of the upper arm, depending on the person’s age (*205*). A needle length appropriate for the person’s age and size should be used (*205*). When HepA vaccines are administered concomitantly with other vaccines or immune globulin (IG), they should be administered in a different anatomic site (e.g., separate limbs) (*205*). If HepA vaccine was administered subcutaneously, inadvertently or for a clinical reason, the dose is considered valid and does not need to be repeated (*205*–*207*).

### Vaccination Schedule and Dosage

#### Vaqta

Vaqta is licensed in two formulations. Persons aged 12 months through 18 years should receive 25 units of HAV antigen per dose in a 2-dose schedule; persons aged ≥19 years should receive 50 units per dose in a 2-dose schedule (Table 1).

**TABLE 1:** Vaccines used to prevent hepatitis A virus infection

Vaccine	Trade name (manufacturer)	Age group (yrs)	Dosage	Route	Schedule	Booster
HepA, inactivated (2 doses)	Havrix (GlaxoSmithKline)	1–18	0.5 mL (720 ELISA units inactivated HAV)	IM	0, 6–12 months	None
		≥19	1 mL (1,440 ELISA units inactivated HAV)	IM	0, 6–12 months	None
HepA, inactivated (2 doses)	Vaqta (Merck)	1–18	0.5 mL (25 units HAV antigen)	IM	0, 6–18 months	None
		≥19	1 mL (50 units HAV antigen)	IM	0, 6–18 months	None
Combined HepA and HepB* (3 doses)	Twinrix (GlaxoSmithKline)	≥18 (primary)	1 mL (720 ELISA units inactivated HAV + 20 *μ*g HBsAg)	IM	0, 1, 6 months	None
		≥18 (accelerated)	1 mL (720 ELISA units inactivated HAV + 20 *μ*g HBsAg)	IM	0, 7, 21–30 days	12 months

**Abbreviations:** ELISA = enzyme-linked immunosorbent assay; HAV = hepatitis A virus; HBsAg = hepatitis B surface antigen; HepA = hepatitis A; HepB = hepatitis B; IM = intramuscular.

* Combined HepA and HepB vaccine (Twinrix) should not be used for postexposure prophylaxis.

#### Havrix

Havrix also is licensed in two formulations. Persons aged 12 months through 18 years should receive 720 ELISA units per dose in a 2-dose schedule; persons aged ≥19 years should receive 1,440 ELISA units per dose in a 2-dose schedule (Table 1).*

#### Twinrix

Twinrix is licensed for use in adults aged ≥18 years. Twinrix contains 720 ELISA units of HAV antigen (half of the Havrix adult dose) and 20 *μ*g of recombinant HBV surface antigen protein (the same as the Engerix-B adult dose) (Table 1). Primary vaccination with Twinrix consists of 3 doses, administered on a 0-, 1-, and 6-month schedule, the same schedule as is commonly used for single-antigen HepB vaccine. After 3 doses of Twinrix, antibody responses to both HAV antigen and HBV surface antigens are equivalent to responses seen after the single-antigen vaccines are administered separately on standard schedules (*208*,*209*); additional information is available in the Twinrix package insert (*202*). Twinrix may be administered before travel or any other potential exposure on an accelerated schedule of 3 doses at 0, 7, and 21–30 days, followed by a booster dose at 12 months that provides long-term protection (*202*). 

#### Booster and Challenge Doses

A HepA vaccine dose may be used to determine the presence of vaccine-induced immunologic memory through generation of an anamnestic response. The term “booster dose” or “challenge dose” has been used to refer to a dose of HepA vaccine administered after a primary vaccination series to provide rapid protective immunity against substantial infection (i.e., infection resulting in serologic test results positive for HAV, clinically significant disease, or both).

### Vaccine Performance

#### Detection of Anti-HAV after Vaccination

**Correlate of protection.** The correlate of protection is defined as an immune response (i.e., IgG anti-HAV titer) that is responsible for and statistically interrelated with protection (*210*). Anti-HAV levels are measured in comparison with a World Health Organization reference IG reagent and are expressed as milli-international units per milliliter (mIU/mL). Seroconversion (response to vaccination) is defined as achieving a detectable and quantifiable postvaccination IgG anti-HAV level of ≥10 mIU/mL by standard assays (*210*,*211*). Seroprotection is considered a surrogate of clinical protection and persists when IgG anti-HAV levels remain higher than the correlate of protection.

The absolute lower limit of anti-HAV needed to prevent HAV infection has not been determined, although the limit is likely quite low because IG provides about 90% efficacy in preventing hepatitis A, and very low levels of anti-HAV are detected in IG recipients (*212*,*213*). The concentrations of anti-HAV achieved after passive transfer by IG or active induction by vaccination are tenfold to 100-fold lower than those produced in response to natural infection (*63*). Clinical studies have yielded limited data from which a minimum protective antibody level can be derived because vaccine-induced levels of antibody are usually high and few infections have been detected among vaccinated persons (i.e., vaccine failure). Antibody levels of 10–33 mIU/mL using different assays have been proposed as the threshold for protection from HAV infection in humans (*214*), although one study proposed that any detectable level of IgG anti-HAV might suggest protection (*72*). Because no absolute protective level has been defined, the lower limit of detection of the particular assay being used has generally typically been considered the protective level; postvaccination studies use ≥10 mIU/mL as the minimum protective level (*210*,*211*,*215*).

**IgM anti-HAV after vaccination.** HepA vaccination can induce detectable IgM anti-HAV, particularly if the test is conducted within a few weeks after vaccination. IgM anti-HAV has been detected 2–3 weeks after administration of 1 dose of vaccine in 8%–20% of adults (*216*) (CDC, unpublished data, 1995). To reduce false-positive tests, persons should only be tested for IgM anti-HAV if they are symptomatic and suspected of having HAV infection (*66*–*68*).

#### Immune Globulin

IG provides protection against hepatitis A through passive transfer of antibody. GamaSTAN is a sterile, preservative-free solution of IG for intramuscular administration and is used for prophylaxis against diseases caused by HAV, measles, varicella, and rubella viruses (see GamaSTAN package insert) (*217*). GamaSTAN is the only IG product approved by FDA for hepatitis A prophylaxis. In 2017, the dosing of IG was changed to reflect decreased IgG anti-HAV potency (*218*,*219*), likely resulting from decreasing prevalence of previous HAV infection among plasma donors (Table 2). GamaSTAN can be administered simultaneously with inactivated vaccines or toxoids in a different anatomic site (e.g., separate limbs) or at any time interval between doses (*205*).

**TABLE 2:** Dosage recommendations for GamaSTAN human immune globulin for preexposure and postexposure prophylaxis against hepatitis A infection

Indication	Time	Dose*	Route
Preexposure prophylaxis	Up to 1 month duration of travel	0.1 mL/kg	IM
Preexposure prophylaxis	Up to 2 months duration of travel	0.2 mL/kg	IM
Preexposure prophylaxis	≥2 months duration of travel	0.2 mL/kg (repeat every 2 months)	IM
Postexposure prophylaxis	Within 2 weeks of exposure	0.1 mL/kg	IM

**Abbreviation:** IM = intramuscular.

*The dosage of immune globulin is based on weight for all ages and does not have a maximum dose for protection against hepatitis A (**Source:** Grifols. Treating with GamaSTAN (immune globulin [human]). https://www.hypermunes.com/en/hcp/gamastan-hepatitis-a).

However, the effect of IG preparations on the response to certain live-virus vaccines is unknown, and antibodies in GamaSTAN might interfere with live-virus vaccines such as measles, mumps, and rubella (MMR) vaccine and varicella vaccine (*205*). When MMR and varicella vaccines are recommended, they should be administered at least 2 weeks before (*205*,*218*) or at least 6 months after the administration of hepatitis A IG (*217*); the GamaSTAN package insert includes additional information (*217*). If an IG preparation must be administered <2 weeks after the administration of MMR or varicella vaccine, the patient should be revaccinated no sooner than 6 months after receipt of the IG preparation (*205*,*217*).

#### Vaccine-Induced Seroprotection

##### Immunogenicity in Infants

Available data indicate that inactivated HepA vaccines are immunogenic in children aged <2 years who do not have passively acquired maternal antibodies (see Factors Associated with Reduced Immunogenicity). All such children administered HepA vaccine subsequently had protective antibody levels, with the final geometric mean concentration (GMCs) varying depending on the dose and schedule (*220*–*226*). Infants (aged <12 months) with passively acquired maternal antibodies had reduced GMCs after vaccination (*221*,*222*).

##### Immunogenicity in Children and Adolescents

HepA vaccines are highly immunogenic when administered to children and adolescents, according to studies using different formulations and vaccine schedules. A total of 97%–100% of persons aged 2–18 years had protective levels of antibody 1 month after receiving the first dose, and 100% had protective levels 1 month after the second dose, with high GMCs (*215*,*225*,*227*–*233*).

##### Immunogenicity in Adults

All licensed HepA vaccines are highly immunogenic in adults aged >18 years when administered according to the recommended schedules (*215*,*229*,*230*,*234*,*235*). Limited data are available regarding the timing needed to develop protective antibodies. A retrospective pooled analysis of four 2-dose vaccination studies compared the immunogenicity and safety of the inactivated HepA vaccine in adults aged ≥40 years with adults aged 20–30 years. The immune response was similar among the age groups at 1 month after the first dose (91%–99.7%) and second dose (95.3%–100%). However, the seroconversion rate was higher at 15 days among adults aged 20–30 years (92.3%; 95% CI: 84–97) than among adults ≥40 years (79.7%; 95% CI: 68.8–88.2) (*234*). For Twinrix, the percentage of persons with seroconversion for hepatitis A was 93.8% after 1 dose, 98.8% after 2 doses, and 99.9% 1 month after 3 doses, using the standard Twinrix schedule; the Twinrix package insert provides additional information (*202*).

##### Efficacy

The efficacy of Havrix was evaluated in a double-blind, randomized controlled clinical trial conducted in Thailand among approximately 40,000 children aged 1–16 years. The children were living in villages with rates of HAV infection of 119 cases per 100,000 population, determined by active surveillance during 1989–1991 (*236*). After 2 doses of vaccine (360 ELISA units per dose) administered 1 month apart, the efficacy of vaccine in protecting against clinical hepatitis A was 94% (95% CI: 79%–99%). A double-blind, placebo-controlled, randomized clinical trial using Vaqta was conducted among approximately 1,000 children aged 2–16 years living in a New York community where 68% percent of persons aged >19 years had detectable levels of antibody (*237*). The protective efficacy against clinical hepatitis A was 100% (lower bound of the 95% CI: 87%) after administration of 1 dose (25 units) of vaccine. The efficacy of Twinrix is expected to be similar to the efficacy for each of the monovalent HepA and HepB vaccine components; the Twinrix package insert provides additional information (*202*).

##### Long-Term Protection

Lifelong protection occurs after natural HAV infection and might occur after vaccination, although the exact duration of protection against HAV infection after vaccination is unknown. Long-term immunogenicity studies have been performed among children vaccinated as children, adults vaccinated as children, and adults vaccinated as adults.

**Children vaccinated at age <24 months.** Anti-HAV seropositivity persisted until at least ages 15–16 years among three groups of Alaska Native children for whom a 2-dose inactivated HepA vaccination series was initiated at ages 6–21 months (*238*). However, among vaccinated infants and children whose mothers were anti-HAV–positive, the prevalence of seropositivity decreased after age 10 years, possibly because of the effect of passively acquired maternal antibodies on immune response. Modeling suggested that seropositivity should persist for most persons for at least 30 years (*238*).

**Adults vaccinated as children.** Anti-HAV has been shown to persist above protective levels for at least 22 years in the majority of adults administered inactivated vaccine on a 3-dose schedule as children (aged 3–6 years) (*239*,*240*), although the overall GMC decreased to 90 mIU/mL, and 12% of participants had anti-HAV levels of <20 mIU/mL, indicating waning anti-HAV (*240*). Vaccine recipients vaccinated as children with the previously recommended 3-dose HepA Havrix (360 ELISA units per dose) schedule were found to have similar protection approximately 14 years after vaccination to those vaccinated with the 2-dose schedule (720 ELISA units) (*241*).

**Adults vaccinated as adults.** Antibody persistence was measured annually in two randomized double-blind studies of adults who received Havrix 1440 ELISA units with a 2-dose schedule of 0–6 months or 0–12 months in 1992–1993 (*242*,*243*). Twenty years after vaccination >97% of adults were seropositive for anti-HAV antibodies. GMCs were 312 mIU/ml in 34 of 36 participants vaccinated initially with a 2-dose schedule at 0–6 months and 317 mIU/ml in 85 of 86 subjects vaccinated at 0–12 months. During 20 years of follow-up, six of seven participants who lost circulating anti-HAV antibodies mounted a strong response after HepA vaccine booster administration. Mathematical modeling predicted that seropositive anti-HAV levels would persist in ≥95% of vaccinees at year 30 and ≥90% at year 40 (*243*).

Periodic assessments of duration of protection continue to be conducted. Although evidence of waning anti-HAV exists, anamnestic response to HepA booster doses indicates persistent immune memory and protection against HAV infection (*144*,*243*). Cellular immunity also might contribute to long-term protection.

##### Single-Dose Hepatitis A Vaccine Protection

Protective anti-HAV antibody levels after a single dose of inactivated HepA vaccine can persist for almost 11 years and increase or reappear after booster vaccination among studies assessing long-term protection up to 10.67 years after a 1-dose vaccination (*244*). In addition, a single dose of HepA vaccine has been shown to induce HAV-specific cellular immunity similar to that induced by natural infection (*245*). A single-dose of single-antigen HepA vaccine has also been shown to control outbreaks of hepatitis A (*246*).

Universal single-dose childhood vaccination programs have been initiated in some South American countries. In 2014, single-dose universal vaccination was adopted by the National Immunization Program of Brazil for children aged 15–24 months (*247*). A significant decrease in HAV cases occurred among children aged <5 years during 2014–2017, from 949 cases to 31 cases (p = 0.017) (*248*). Single-dose HepA vaccination was implemented for all Argentinean children at age 12 months in 2005. Protective antibody levels against HAV were found in more than 97% (1,060 of 1,088) of Argentinean children up to age 9 years after single-dose HepA vaccination (*249*) and in approximately 87.6% of 1,119 children tested up to 11 years after single-dose vaccination (*250*). Additional data are needed to assess long-term protection after a single dose.

#### Factors Associated with Reduced Immunogenicity

##### Presence of Passively Acquired Maternal Antibodies

The presence of passively acquired maternal anti-HAV at the time of infant (aged <12 months) vaccination interferes with the immune response to HepA vaccination and substantially reduces the anti-HAV concentration after vaccination (*220*,*221*,*224*,*251*). In the majority of studies, although all infants subsequently had protective levels of antibody, the final GMCs were approximately one third to one tenth the GMCs of infants born to anti-HAV–negative mothers and vaccinated according to a single, consistent schedule (*252*). Despite lower antibody levels after the primary series, the majority of infants with passively acquired antibody had an anamnestic response to a booster dose administered 1–6 years later (*221*,*252*,*253*). Passively acquired antibody decreased to undetectable levels in the majority of infants by age 1 year (*220*,*254*). HepA vaccine is highly immunogenic for children who begin vaccination at age >1 year, regardless of maternal anti-HAV status (*220*,*221*).

##### Concurrent Administration of Hepatitis A Vaccine and Immune Globulin

Although GMCs of adults who received IG and vaccine were lower 1 month after completion of the vaccine series than the GMCs of adults who had been administered HepA vaccine alone, the proportion of adults who subsequently had protective levels of antibody did not differ (*255*,*256*). Therefore, the effect of reduced GMCs on long-term protection is unknown.

##### Immunocompromising Conditions

The humoral response to HepA vaccine might be reduced in immunocompromised children and adults (e.g., hematopoietic cell transplant [HCT] recipients, patients undergoing chemotherapy, and persons with HIV infection) (Table 3) (*4*,*257*–*259*). Limited data suggest that modified dosing regimens, including a doubling of the standard antigen dose or administration of additional doses, might increase response rates. In addition, ACIP best practice guidance states that HCT recipients who received vaccines before their HCT should be vaccinated or revaccinated routinely after HCT, regardless of the source of the transplanted stem cells; revaccination doses of HepA vaccine are recommended after HCT (*205*).

**TABLE 3:** Categories of persons with increased risk for hepatitis A virus infection or severe disease from hepatitis A virus infection*

Type of risk	Risk category	Examples
Increased risk for HAV infection	Close personal contacts of persons with HAV infection^†^	Household contacts
		Caretakers
		Sexual contacts
		Persons who anticipate close personal contact with an international adoptee
	Occupational risk	Persons working with nonhuman primates
		Persons working with clinical or nonclinical material containing HAV in a research laboratory
	Persons who use drugs	Persons who use injection or noninjection drugs (i.e., all those who use illegal drugs)
	Persons in settings where services to adults are provided	Group settings for persons with developmental disabilities
		Homeless shelters
		Syringe services programs
		Correctional facilities during outbreaks
	International travelers	Persons traveling to or working in countries with high or intermediate HAV endemicity
Increased risk for severe disease from HAV infection	Immunocompromised persons	Congenital or acquired immunodeficiency
		HIV infection
		Chronic renal failure, undergoing dialysis
		Solid organ, bone marrow, or stem cell transplant recipients
		Persons with diseases requiring treatment with immunosuppressive drugs/biologics (e.g., tumor necrosis alpha inhibitors), long-term systemic corticosteroids, radiation therapy
	Persons with chronic liver disease	Hepatitis B virus infection
		Hepatitis C virus infection
		Cirrhosis (any etiology)
		Fatty liver disease (hepatic steatosis)
		Alcoholic liver disease
		Autoimmune hepatitis
		Alanine aminotransferase or aspartate amino transferase level more than twice the upper limit of normal or persistently elevated for 6 months
	Age	Adults aged >40 years

**Abbreviations:** HAV = hepatitis A virus; HIV = human immunodeficiency virus.

* Not all risk categories include persons recommended for routine hepatitis A vaccination (Box). Providers should assess the risk for HAV infection or severe disease from HAV infection when making decisions regarding the provision of postexposure prophylaxis or revaccination (Table 4). Providers should consider vaccination in settings providing services to adults at risk for HAV infection (see Implementation Strategies and Hepatitis A Vaccination During Outbreaks).

^†^ Excludes health care personnel using appropriate personal protective equipment.

The Infectious Disease Society of America has guidance for vaccination of the immunocompromised host. The guidance states that solid organ transplant candidates who are unvaccinated, undervaccinated, or seronegative for hepatitis A, particularly liver transplant candidates, aged 12–23 months (strong recommendation, moderate-quality evidence) and ≥2 years (strong recommendation, moderate-quality evidence) should receive a HepA vaccine series (*4*).

##### Lower CD4 Counts and Other Factors Among Persons with HIV Infection

HepA vaccine using a standard dose and schedule is immunogenic for children and adults with HIV infection (*143*,*144*,*260*,*261*). Although those with higher CD4 counts (>200–300 cells/mm^3^) respond nearly as well as persons who are not immunocompromised, adults with lower CD4 counts are less likely to acquire protective levels of antibody (*139*,*141*,*145*–*147*,*257*,*260*,*262*). This finding suggests that immunologic reconstitution with antivirals might restore the ability to respond to vaccination (*263*). Most adults with well-controlled HIV infections had durable seropositive responses up to 6–10 years after HepA vaccination (*145*). In one study, protective antibodies developed in 100% of 32 children with HIV infection (mean age: 5.5 years) (*264*). Factors associated with seroreversion (loss of seroresponse) in persons with HIV infection who had an initial seroconversion include higher weight (overweight and obese), lower CD4 count or HIV viremia at the time of HepA vaccination, or lower or delayed seroresponse to vaccination (*146*).

##### Chronic Liver Disease

Vaccination of children or adults with chronic liver disease of viral or nonviral etiology produced seroprotection proportions similar to those observed in healthy adults (Table 3) (*265*–*270*). Seroprotection in liver transplant recipients is highly variable. In one study, none of the eight patients who were vaccinated after liver transplantation responded to HepA vaccination; in another study, six (26%) of 23 liver transplant recipients responded (*265*,*266*). However, HepA vaccine was immunogenic for most liver transplant patients in another study, with 38 (97%) responding to a standard dose and schedule (*271*,*272*) (see Immunocompromising Conditions).

##### Older Age (Aged >40 Years)

Limited data suggest that persons vaccinated at an older age might have a lower immune response to HepA vaccine than younger persons. In certain studies, persons vaccinated at age >40 years had lower rates of seroprotection than persons vaccinated at age ≤40 years, particularly when levels were measured within 15 days of the first dose, but had similar seroprotection rates after two doses, despite lower final antibody levels (*229**,**234**,**235*,*273*–*275*). 

##### Other Factors

A slower antibody response to HepA vaccine has been observed in overweight persons (*146*,*273*), and one study among persons with HIV infection found that seroreversion was more likely when persons were overweight or obese (*146*). Smoking, a factor associated with decreased immunogenicity to other vaccines, has not been evaluated for the licensed formulations of HepA vaccine.

#### Vaccine Safety

##### Administration of Hepatitis A Single-Antigen and Combination Vaccines

In prelicensure clinical trials, the most common adverse events following vaccination with Havrix and Vaqta (HepA) and Twinrix (combination HepA and HepB) were injection site reactions (e.g., pain and erythema) and mild systemic reactions (e.g., fever, irritability, loss of appetite, drowsiness, and headache). Rates of adverse events following Twinrix vaccination were similar to those observed with separately administered HepA and HepB vaccines.

VAERS, a spontaneous reporting (passive surveillance) system managed by CDC and FDA, is used to conduct post-licensure safety monitoring of U.S. vaccines (*18*). During 2006–2018, VAERS received 25,079 U.S. reports involving HepA vaccines, of which 5.7% were classified as serious (i.e., one or more of the following was reported: death, life-threatening illness, hospitalization or prolongation of existing hospitalization, or permanent disability) (*276*). Among all reports, 48.6% were in persons aged 2–18 years, 29.5% in children aged 12–23 months, and 13.3% in adults aged >19 years. Most (80.3%) reports involved HepA vaccines administered concomitantly with other vaccines during the same health care visit. The most frequently reported adverse events were fever (16.2%), injection site erythema (14.8%), injection site swelling (9.9%), rash (9.0%), and erythema (8.9%) (CDC, unpublished data, 2006–2018).

During the same period (2006–2018), VAERS received 2,117 U.S. reports involving the combination HepA and HepB vaccine, of which 8.6% were classified as serious. Among all reports, 92.9% were in persons aged ≥18 years (the approved age for the licensed vaccine). A majority (60.3%) of reports involved combination Twinrix vaccine administered concomitantly with other vaccines during the same health care visit. The most frequently reported adverse events were fever (13.6%), headache (11.5%), pain (11.5), injection site pain (9.8%), and dizziness (9.4%) (CDC, unpublished data, 2006–2018).

VAERS is subject to the limitations of spontaneous reporting systems in general and is not designed to assess whether a vaccine caused an adverse event. However, these findings in VAERS for HepA vaccine and combination Twinrix vaccine are similar to findings for other inactivated vaccines routinely administered in these age groups.

The data on HepA vaccination during pregnancy are limited. A published safety review of 139 reports to VAERS during 1996–2013 of women who received HepA vaccine or combination Twinrix vaccine while pregnant did not identify any concerning patterns of adverse events in the pregnant women or their infants (*277*).

A multisite study (*278*) in CDC’s Vaccine Safety Datalink (VSD), a population-based research and surveillance data system (*20*), of maternal HepA vaccination found that HepA vaccine administration during pregnancy was not associated with increased risk for a range of adverse events examined among pregnancies resulting in live births. However, an association was found between maternal HepA vaccination and infants who were small for gestational age. Investigators believe this association was likely due to unmeasured confounding but might warrant additional consideration (*278*).

Persons who have had a severe allergic reaction (e.g., anaphylaxis) after a previous dose of HepA vaccine or have severe allergy to a HepA vaccine component should not receive HepA vaccine (*205*). Persons who have a moderate or severe acute illness with or without fever might want to defer vaccination while the acute illness is present (*205*).

##### Simultaneous Administration with Other Vaccines

Available data do not indicate a reduced response when HepA vaccine is administered with other vaccines among children and adults (*221*,*279*–*285*); additional information is available from the manufacturer package inserts (*202*). Limited data from studies conducted among adults indicate that simultaneous administration of HepA vaccine with diphtheria, poliovirus (oral and inactivated), tetanus, typhoid (both oral and intramuscular), cholera, Japanese encephalitis, rabies, or yellow fever vaccines does not decrease the immune response to either vaccine or increase the frequency of adverse events (*279*–*281*). Studies conducted before the licensure of Twinrix in the United States indicated that HepB vaccine can be administered simultaneously with HepA vaccine without either decreasing vaccine immunogenicity or increasing the frequency of adverse events (*282*,*283*).

Studies conducted among infants and young children aged <18 months have demonstrated that simultaneous administration of HepA vaccine with diphtheria-tetanus-acellular pertussis (DTaP), *Haemophilus influenzae* type b (Hib), HepB, MMR, or inactivated poliovirus vaccines does not affect the immunogenicity and reactogenicity of these vaccines (*202*,*221*,*283*,*284*). Administration of 1 or 2 doses of Vaqta was well tolerated and highly immunogenic in approximately 4,300 children aged 12–23 months, whether given alone or concomitantly with other vaccines (M-M-RII, Varivax, Tripedia, Prevnar, ProQuad, PedvaxHIB, and Infanrix) in the United States (*285*).

#### Cost-Effectiveness Considerations

The cost-effectiveness of nationwide routine HepA vaccination was evaluated in an analysis published in 2007 that used a Markov model to follow a single U.S. birth cohort of approximately 4 million persons from birth in 2005 through age 95 years or death (*286*). Compared with no vaccination, routine vaccination at age 1 year would prevent 172,000 infections at a cost of $28,000 per quality-adjusted life year (QALY) saved. Compared with maintaining the levels of HepA vaccination under the preexisting regional policy in the 1996 and 1999 ACIP recommendations (*6*,*7*), universal routine vaccination at age 1 year would prevent an additional 112,000 infections, at a cost of $45,000 per QALY saved (*286*). Another economic analysis that considered the estimated reduction in secondary cases among household contacts of infected children yielded similar results (*287*).

After implementation of the 2006 universal HepA childhood vaccination recommendations (*8*), in 2015 the original Markov model was adapted to assess the cost-effectiveness of catch-up HepA vaccination among unvaccinated and partially vaccinated children compared with unvaccinated children, using hepatitis A incidence rates during 2008–2012 (*288*). Over the lifetime of the cohort, catch-up HepA vaccination would reduce the total number of infections relative to the baseline by 741 while increasing doses of vaccine administered by 556,989. Catch-up vaccination would increase net cost by $10.2 million, or $2.38 per person (*288*). The model’s conclusions were highly sensitive to the discount rate (3%), rate of adult vaccination, and the incidence of HAV infection. Although vaccination rates among adults have persisted at low levels (*289*), the hepatitis A incidence rate and child and adolescent vaccination rates are substantially higher than when this model was developed (*9*,*14*). Therefore, the actual cost-effectiveness of catch-up vaccination is likely more favorable now than originally estimated.

## Recommendations for Hepatitis A Vaccine and Immune Globulin for Preexposure Prophylaxis

This section contains ACIP recommendations for routine HepA vaccination among children, adolescents, and adults (Box) and guidance for the prevention of HAV infection using IG for preexposure and postexposure prophylaxis. HepA vaccination is also recommended by ACIP for postexposure prophylaxis and for persons at risk during outbreaks. IG provides short-term protection against HAV infection and is recommended in certain situations (Tables 2 and 4).

BOX Prevention of hepatitis A virus infection in the United States: Recommendations of the Advisory Committee on Immunization Practices, 2020*

**Children**

All children aged 12–23 months

Unvaccinated children and adolescents aged 2–18 years

**Persons at increased risk for HAV infection**

International travelers

Men who have sex with men

Persons who use injection or noninjection drugs (i.e., all those who use illegal drugs)

Persons with occupational risk for exposure

Persons who anticipate close personal contact with an international adoptee

Persons experiencing homelessness

**Persons at increased risk for severe disease from HAV infection**

Persons with chronic liver disease

Persons with human immunodeficiency virus infection

**Other persons recommended for vaccination**

Pregnant women at risk for HAV infection or severe outcome from HAV infection

Any person who requests vaccination

**Vaccination during outbreaks**

Unvaccinated persons in outbreak settings who are at risk for HAV infection or at risk for severe disease from HAV

**Implementation strategies for settings providing services to adults**

Persons in settings that provide services to adults in which a high proportion of those persons have risk factors for HAV infection

**Hepatitis A vaccination is no longer recommended by ACIP**

Persons who receive blood products for clotting disorders (e.g., hemophilia)

*See the Recommendations for Hepatitis A Vaccine and Immune Globulin for Preexposure Prophylaxis section in this report for additional information.

**TABLE 4:** Recommendations for postexposure prophylaxis and preexposure protection, by age group and risk category — Advisory Committee on Immunization Practices, 2020

Indication and age group	Risk category and health status	HepA vaccine	IG*
**Postexposure prophylaxis**			
<12 months	Healthy	No	0.1 mL/kg
12 months–40 yrs	Healthy	1 dose^†^	None
>40 yrs	Healthy	1 dose^†^	0.1 mL/kg^§^
≥12 months	Immunocompromised or chronic liver disease	1 dose^†^	0.1 mL/kg^¶^
≥12 months	Vaccine contraindicated**	No	0.1 mL/kg
**Preexposure protection (e.g., travel)** ^††^			
<6 months	Healthy	No	0.1–0.2 mL/kg^§§^
6–11 months	Healthy	1 dose^¶¶^	None
12 months–40 yrs	Healthy	1 dose***	None
>40 yrs	Healthy	1 dose***	0.1–0.2 mL/kg^§§,†††^
>6 months	Immunocompromised or chronic liver disease	1 dose***	0.1–0.2 mL/kg^§§,†††^
>6 months	Persons who elect not to receive vaccine or for whom vaccine is contraindicated**	No	0.1–0.2 mL/kg^§§^

**Abbreviations:** HAV = hepatitis A virus; HepA = hepatitis A; IG = immune globulin.

* Measles, mumps, and rubella vaccine should not be administered for at least 2 weeks before and 6 months after administration of IG.

^†^ A second dose of HepA vaccine is not required for postexposure prophylaxis; however, for long-term immunity, the vaccination series should be completed with a second dose at least 6 months after the first dose.

^§^ The provider’s risk assessment (Table 3) should determine the need for IG administration. If the provider’s risk assessment determines that both vaccine and IG are warranted, HepA vaccine and IG should be administered simultaneously in a different anatomic site (e.g., separate limbs).

^¶^ Vaccine and IG should be administered simultaneously in a different anatomic site (e.g., separate limbs).

** Life-threatening allergic reaction to a previous dose of HepA vaccine or allergy to any vaccine component.

^††^ IG should be considered before travel for persons with special risk factors for either HAV infection or severe disease from HAV infection.

^§§^ 0.1 mL/kg for travel up to 1 month; 0.2 mL/kg for travel up to 2 months, 0.2mL/kg every 2 months for travel of ≥2 months’ duration.

^¶¶^ This dose should not be counted toward the routine 2-dose series, which should be initiated at age 12 months.

*** For persons not previously vaccinated with HepA vaccine, administer dose as soon as travel is considered, and complete series according to routine schedule if the next dose is needed before travel.

^†††^ May be administered based on provider’s risk assessment (Table 3).

The following recommendations for HepA vaccination are intended to further reduce hepatitis A morbidity and mortality in the United States and make possible consideration of eventual elimination of HAV transmission. HepA vaccination is recommended routinely for children, persons at increased risk for infection, persons at increased risk for severe disease from HAV infection, and any person who requests vaccination.

### Children

- ACIP recommends Hep A vaccination for all children aged 12–23 months,
- ACIP recommends that all children and adolescents aged 2–18 years who have not previously received HepA vaccine be vaccinated (i.e., children and adolescents are recommended for catch-up vaccination). This is a new recommendation.

### Persons at Increased Risk for HAV Infection

#### Persons Traveling to or Working in Countries with High or Intermediate HAV Endemicity 

All susceptible persons (i.e., unvaccinated, partially vaccinated, or never infected) traveling to or working in countries that have high or intermediate hepatitis A endemicity (Figure 3) are at increased risk for HAV infection. These persons should be vaccinated, or receive IG if too young or contraindicated for vaccine, before departure (*17*,*218*). For travelers who are partially vaccinated already (i.e., did not receive a full vaccine series), a dose should be administered before travel if needed according to the vaccine schedule.

##### Infants

**Infants aged <6 months.** Infants aged <6 months should receive IG before travel when protection against HAV is recommended. For travel duration up to 1 month, 1 dose of IG at 0.1 mL/kg is recommended; for travel up to 2 months, 1 dose of IG at 0.2 mL/kg is recommended, and for travel of ≥2 months, a 0.2 mL/kg dose of IG should be repeated every 2 months for the duration of travel or until the infant is administered HepA vaccine (i.e., at age ≥6 months) (Tables 2 and 4).

**Infants aged 6–11 months.** HepA vaccine should be administered to infants aged 6–11 months traveling outside the United States when protection against HAV is recommended (Table 4). The travel-related dose for infants aged 6–11 months does not count toward the routine 2-dose series. Therefore, the 2-dose HepA vaccination series should be initiated at age 1 year with the appropriate dose and schedule.

**CDC clinical guidance.** IG (GamaSTAN) cannot be administered simultaneously with MMR vaccine because antibody-containing products such as IG can inhibit the immune response to measles and rubella vaccines for up to 6 months after administration (*217*). However, because MMR vaccine is recommended for all infants aged 6–11 months traveling internationally from the United States (*290*), and because measles in infancy is more severe than HAV infection in infancy, measles prevention should be prioritized, and IG should not be administered. HepA vaccine (indication for off-label use) and MMR vaccine can be administered simultaneously to infants aged 6–11 months (*220*,*221*,*251*), providing protection against both hepatitis A and measles (*17*,*205*,*284*).

##### Persons Aged ≥12 Months

**Healthy persons aged 12 months through 40 years.** Healthy persons aged 12 months through 40 years who are planning on traveling to an area with high or intermediate hepatitis A endemicity and who have not received HepA vaccine should receive a single dose of HepA vaccine as soon as travel is considered and should complete the HepA vaccine series with the appropriate dose and schedule (Table 4).

**Persons aged >40 years, persons with immunocompromising conditions, and persons with chronic liver disease.** Persons aged >40 years, persons with immunocompromising conditions, and persons with chronic liver disease planning on traveling to an area with high or intermediate HAV endemicity should receive a single dose of HepA vaccine as soon as travel is considered. Persons traveling in <2 weeks should receive the initial dose of HepA vaccine and simultaneously may be administered IG in a different anatomic injection site (e.g., separate limbs) (*218*,*268*,*269*,*291*). The HepA vaccine series should be completed according to the routine schedule (Table 4).

**CDC clinical guidance.** In addition to HepA vaccine, IG should be considered before travel for persons with special risk factors or increased risk for severe disease from HAV infection (Table 3) (Appendix A).

**Travelers for whom vaccine is contraindicated and persons who choose not to receive HepA vaccine.** Travelers for whom vaccine is contraindicated or who choose not to receive vaccine should receive IG before travel when protection against HAV is recommended. For travel duration up to 1 month, 1 dose of IG at 0.1 mL/kg is recommended; for travel up to 2 months, 1 dose of IG at 0.2 mL/kg is recommended, and for travel of ≥2 months, a 0.2 mL/kg dose of IG should be repeated every 2 months for the duration of travel (Tables 2 and 4).

#### Men Who Have Sex with Men

Vaccination is recommended for MSM. Health care providers in primary care and specialty medical settings in which MSM receive care (e.g., sexually transmitted disease clinics) should offer HepA vaccine to all MSM.

#### Persons Who Use Injection or Noninjection Drugs

Vaccination is recommended for persons who use injection or noninjection drugs (i.e., all those who use illegal drugs). Health care providers should conduct a thorough risk factor assessment to identify persons who use or are at risk for using injection or noninjection drugs. Health care providers in primary care and specialty medical settings in which persons who use drugs receive care (e.g., opioid treatment programs that provide medication-assisted treatment to persons with an opioid use disorder) should offer HepA vaccine to all persons who use injection or noninjection drugs.

#### Persons with Occupational Risk for Exposure

Persons who work with HAV-infected nonhuman primates or with clinical or nonclinical material containing HAV in a research laboratory setting should be vaccinated. No other occupational groups (e.g., health care providers or food handlers) have been demonstrated to be at increased risk for HAV infection because of occupational exposure.

#### Persons Who Anticipate Close Personal Contact with an International Adoptee from a Country with High or Intermediate Endemicity

All persons who anticipate close personal contact (e.g., household contact, caretaker, or regular babysitter) with an international adoptee from a country with high or intermediate endemicity during the first 60 days following arrival of the adoptee in the United States should be vaccinated against hepatitis A (*16*). The first dose of the HepA vaccine series should be administered as soon as adoption is planned, ideally 2 or more weeks before the arrival of the adoptee.

#### Persons Experiencing Homelessness

All persons aged ≥1 year experiencing homelessness should be routinely vaccinated against hepatitis A. HepA vaccine should be integrated into routine preventive services for persons experiencing homelessness (*15*).

#### Persons at Increased Risk for Severe Disease from HAV Infection

##### Persons with Chronic Liver Disease

Persons with chronic liver disease (including but not limited to persons with HBV infection, HCV infection, cirrhosis, fatty liver disease, alcoholic liver disease, autoimmune hepatitis, or an ALT or AST level persistently greater than twice the upper limit of normal) should be routinely vaccinated against hepatitis A.

##### Persons with HIV Infection

**Persons aged ≥1 year.** ACIP recommends all persons with HIV infection aged ≥1 year be routinely vaccinated with HepA vaccine. This is a new recommendation.

**CDC clinical guidance.** Because the response to the vaccine might be reduced in persons with HIV infection who are immunosuppressed, postvaccination serologic testing should be performed for all persons with HIV infection ≥1 month after completing the HepA vaccine series (*292*). Some studies have shown that ≥6 months is needed for persons with HIV infection to seroconvert (i.e., ≥10 mIU/mL) after vaccination (*146*,*257*,*293*,*294*).

Although persons with HIV infection who have lower CD4 cell counts or percentages might have a weaker response to the vaccine, vaccination should not be delayed until the CD4 count exceeds a certain threshold because of the prolonged risk for HAV exposure created by missed opportunities to vaccinate. Persons with HIV infection who do not respond to the vaccine should be considered susceptible to HAV infection and counseled about precautions to prevent infection, as well as the need to obtain IG postexposure prophylaxis (PEP) for any known or likely exposure to HAV. Health care providers should consider revaccination (repeat vaccine series) for persons with HIV infection who did not demonstrate an adequate immune response (i.e., ≥10 mIU/mL) after the initial HepA vaccination series, particularly persons with HIV infection who later demonstrate improvement in immune status (e.g., increased CD4 counts and decreased HIV viral load) (*292*). At least 1 month after revaccination with a complete vaccine series, postvaccination testing should be performed. If the response to revaccination is still not adequate (i.e., <10 mIU/mL), additional vaccination is not recommended; however, the person should be counseled concerning methods to prevent HAV infection, including the need for IG after an exposure (see Revaccination).

All persons with HIV infection who received HepA vaccine, regardless of postvaccination serologic testing results, should be counseled that the vaccine might not provide long-term protection against HAV infection. Therefore, they might need to receive IG after a high-risk HAV exposure (e.g., a sexual or household contact) (*146*,*295*).

#### Other Persons Recommended for Vaccination

##### Pregnant Women

**Pregnant women at risk for HAV infection or severe outcome from HAV infection.** ACIP recommends that pregnant women who are identified to be at risk for HAV infection during pregnancy (e.g., international travelers, persons who use injection or noninjection drugs, persons who have occupational risk for infection, persons who anticipate close personal contact with an international adoptee, or persons experiencing homelessness) or for having a severe outcome from HAV infection (e.g., persons with chronic liver disease or persons with HIV infection) should be vaccinated during pregnancy if not previously vaccinated. This is a new recommendation.

**CDC clinical guidance.** Pregnant women should be vaccinated for the same indications as nonpregnant women. Unvaccinated or partially vaccinated pregnant adolescents should receive HepA catch-up vaccination. Pregnant women at risk for HAV infection during pregnancy should be counseled concerning other prevention methods (e.g., hand hygiene) to prevent HAV infection.

##### Any Person Who Requests Vaccination

Disclosure of a risk factor for HAV infection or complication is not necessary for HepA vaccination. Because a person might not disclose a risk factor to the provider, ACIP recommends that any person who has not previously completed the HepA vaccine series may receive HepA vaccine.

### Implementation Strategies for Settings Providing Services to Adults

Settings in which a high proportion of persons have risk factors for HAV infection include health care settings that focus on persons who use injection or noninjection drugs, as well as group homes and nonresidential day care facilities for persons with developmental disabilities. Health care providers may assume that unvaccinated adults aged ≥19 years in these settings are at risk for HAV infection and offer HepA vaccination to those who have not previously completed vaccination.

- HepA vaccination may be offered in outreach and other settings in which services are provided to persons at risk for HAV infection (e.g., homeless shelters and syringe services programs).
- Health care providers should consider implementing standing orders to identify adults recommended for HepA vaccination and administer vaccination as part of routine services.
- HepA vaccination should be considered for persons (e.g., residents and staff) in facilities where hygiene is difficult to maintain (e.g., group homes for persons with development disabilities, and homeless shelters.)

### ACIP Recommendations for Hepatitis A Vaccine and Immune Globulin for Postexposure Prophylaxis

Guidance for providers on risk assessment and clinical decision-making for HAV PEP is provided (Appendix B). In the absence of PEP, secondary attack rates of 20%–50% have been reported in households with higher rates of transmission occurring from infected young children than from infected adolescents and adults (*34*,*35*,*296*,*297*). Persons who have been exposed to HAV within the last 2 weeks (e.g., a sexual or household contact or known contaminated food source) and who have not been vaccinated previously should be administered 1 dose of single-antigen HepA vaccine, IG (0.1 mL/kg), or both (persons aged ≥12 months who are immunocompromised or have chronic liver disease) as soon as possible (*17*,*218*). Using HepA vaccine for PEP has advantages over IG, including induction of active immunity, longer duration of protection, ease of administration, and greater acceptability and availability (*17*). The efficacy of IG or vaccine administered >2 weeks after exposure has not been established.

Attack rates among persons exposed to HAV-infected food handlers are low (*171*,*173*); most food handlers with HAV infection do not transmit HAV through the workplace to exposed consumers or restaurant patrons (*36*,*170*,*171*). 

Severely immunocompromised persons, including those who receive routine IG administration for immunocompromising conditions, who have been vaccinated in the past may benefit from PEP and should be assessed on an individual basis for risk for HAV infection. 

HepA vaccine should be administered as soon as possible, within 2 weeks of exposure, to all unvaccinated persons aged ≥12 months who have recently been exposed to HAV (*17*). In addition to HepA vaccine, coadministration of GamaSTAN IG (0.1 mL/kg) is recommended under certain circumstances (Table 4) and for persons aged >40 years based on the provider’s risk assessment (*17*).

#### Infants Aged <12 Months

Infants aged <12 months should receive IG (0.1 mL/kg) (*17*,*218*) instead of HepA vaccine as soon as possible within 2 weeks of exposure. MMR vaccine should not be administered <6 months after IG administration (*217*); the GamaSTAN package insert includes additional information (*217*).

#### Persons Aged ≥12 Months

##### Healthy Persons Aged ≥12 Months

Persons aged ≥12 months who have been exposed to HAV within the past 2 weeks and have not previously completed the HepA vaccine series should receive 1 dose of single-antigen HepA vaccine (Table 4) as soon as possible. In addition to HepA vaccine, IG (0.1 mL/kg) may be administered to persons aged >40 years depending on the provider’s risk assessment (Appendix B). When the dose of HepA vaccine administered for PEP is the first dose the exposed person has received, a second dose should be administered 6 months after the first for long-term immunity; however, the second dose is not necessary for PEP.

##### Persons Aged ≥12 Months Who Are Immunocompromised or Have Chronic Liver Disease

Persons who are immunocompromised or have chronic liver disease and who have been exposed to HAV within the past 2 weeks and have not previously completed the HepA vaccination series should receive both IG (0.1 mL/kg) and HepA vaccine simultaneously in a different anatomic site (e.g., separate limbs) as soon as possible after exposure (*17*,*268*,*269*,*291*) (Table 4). When the dose of HepA vaccine administered for PEP is the first dose the exposed person has received, a second dose should be administered 6 months after the first for long-term immunity; however, the second dose is not necessary for PEP.

##### Persons for Whom Vaccine is Contraindicated

Persons for whom vaccine is contraindicated (see Vaccine Safety) should receive IG (0.1 mL/kg) (*17*,*218*) instead of HepA vaccine as soon as possible within 2 weeks of exposure. MMR and varicella vaccines should not be administered <6 months after IG administration (*217*); the GamaSTAN package insert includes additional information (*217*).

#### CDC Clinical Guidance for Postexposure Prophylaxis

In addition to HepA vaccine, IG should be considered for PEP for persons with special risk factors for either HAV infection or severe disease from HAV infection (Tables 3 and 4) (Appendix B). The combination HepA and HepB vaccine, Twinrix, contains 720 ELISA units of hepatitis A antigen, which is half of the single-antigen HepA vaccine adult dose. Because no data are available for use of Twinrix for PEP, Twinrix is not recommended for use as PEP.

#### Vaccination During Outbreaks

##### Preexposure Vaccination of Populations at Risk

Vaccination strategies for outbreak control should focus on preexposure vaccination of populations at risk for infection and postexposure strategies. The best way to prevent HAV infection is through vaccination of at-risk populations. There is limited available evidence indicating the level of vaccine coverage needed to control a hepatitis A outbreak (*84*,*246*,*298*). For routine vaccination, the number and timing of doses depends on the type of vaccine administered; however, a single dose of HepA vaccine is adequate for outbreak control (*246*). Providers should complete the HepA vaccine series when feasible; however, completion of the series, although recommended for long-term protection, is not required for PEP or an outbreak response.

Certain populations at risk for HAV infection (e.g., persons who use injection or noninjection drugs or persons experiencing homelessness) in an outbreak might be difficult to reach with routine vaccination and education efforts because of various factors (e.g., behavioral health issues, limited or no engagement in the health care system and other institutions, and lack of transportation). Often, health departments and health care providers must supplement traditional outreach strategies with other approaches (e.g., mobile field vaccination, stationary points of dispensary, and vaccination foot teams) to reach these populations (*299*).

ACIP recommends 1 dose of HepA vaccine during a hepatitis A outbreak for all unvaccinated persons aged ≥1 year who are at risk for HAV infection (e.g., persons who use injection or noninjection drugs, persons experiencing homelessness, and MSM) (*12*,*300*) or at risk for severe disease from HAV (e.g., persons with chronic liver disease and persons with HIV infection). A single dose of preexposure HepA vaccine has been shown to successfully control outbreaks of hepatitis A (*246*).

##### CDC Clinical Guidance

In the event of a community outbreak propagated by person-to-person transmission, public health officials should consider recommending administration of preexposure HepA vaccination in close congregate settings providing services to persons at high risk for infection (e.g., persons incarcerated in correctional facilities, health care settings for persons who use injection or noninjection drugs, homeless shelters, and syringe services programs) who are in the vicinity of the outbreak. Increased risk for HAV infection and therefore an increased risk for an outbreak exists in these settings (*12*,*55*,*299*,*301*). Congregate settings offer a unique opportunity to efficiently vaccinate during an outbreak, and expanded vaccination of populations at risk for infection during an outbreak has the potential to halt transmission more quickly than nontargeted approaches (*12*).

#### Serologic Testing

##### Prevaccination

Prevaccination serologic testing for hepatitis A immunity before vaccination is not routinely recommended but may be considered in specific settings to reduce costs by not vaccinating persons who are already immune. Prevaccination serologic testing should not be a barrier to vaccination of susceptible persons, especially in populations that are difficult to access. If prevaccination serologic testing is performed, commercially available tests for total anti-HAV or IgG anti-HAV should be used.

Persons for whom prevaccination testing will likely be most cost-effective include adults who were either born in or lived for extensive periods in geographic areas with high or intermediate hepatitis A endemicity (Figure 3). Prevaccination serologic testing of children is not indicated because of the low prevalence of infection in this age group (*9*).

In populations that are expected to have high rates of previous HAV infection, vaccination history should be obtained when feasible before testing or vaccination. Vaccination should not be postponed if vaccination history cannot be obtained, if records are unavailable, or if prevaccination testing is not feasible. Vaccinating persons immune from natural infection carries no known risk, nor does giving extra doses of HepA vaccine (*302*).

##### Postvaccination

Serologic testing for immunity is not necessary after routine vaccination of infants, children, or adults. Testing for the presence of anti-HAV antibody after vaccination is recommended for persons whose subsequent clinical management depends on knowledge of their immune status and persons for whom revaccination might be indicated, such as persons with HIV infection and other immunocompromised persons (e.g., HCT and solid organ transplant recipients and persons receiving chemotherapy) (see Persons with HIV Infection; Revaccination).

#### Revaccination

Revaccination (i.e., booster dose, challenge dose, or revaccination with a complete series) is not recommended for healthy persons who were vaccinated with a complete series as infants, children, or adults (*238*,*240*,*243*). For immunocompromised persons (e.g., persons with HIV infection, HCT and solid organ transplant recipients, and persons receiving chemotherapy), limited data are available to determine the need for booster doses or revaccination with a complete series.

Clinicians may consider revaccinating persons with HIV infection (see Persons with HIV Infection) and persons who received HepA vaccine while immunosuppressed from chemotherapy who failed to demonstrate an adequate immune response (i.e., ≥10 mIU/mL) after the initial HepA vaccination series (*146*,*205*). In such cases, postvaccination serologic testing should be performed at least 1 month after revaccination with a complete vaccine series. If the response to revaccination is still not adequate (i.e., <10 mIU/mL), additional vaccination is not recommended; however, the person should be counseled concerning methods to prevent HAV infection, including the need for IG after an exposure. If vaccination results in seroconversion, limited data exist on the need for repeat testing or on the frequency of booster doses or revaccination.

#### Interrupted Schedules and Minimum Dosing Intervals

- **Schedule interruption.** For all ages, when the HepA vaccine schedule is interrupted, the vaccine series does not need to be restarted. Limited data indicate that the response to a second dose delayed for 20–31 months in persons age >2 years (Havrix) and delayed 4–8 years among adults (Havrix) was similar to that of the licensed schedules. Among adults, the response to a second dose delayed for 12–18 months (Vaqta) was similar to the response to a booster dose administered after 6 months (*303*–*305*).
- **First and second dose administered.** If the first and second doses of single-antigen HepA vaccine were administered <6 months apart, then the second dose is invalid and should be repeated 6 months after the invalid second dose. However, if this repeat dose (the third dose) is administered any time ≥6 months after the first dose and at least the age-appropriate dose was administered, the series can be considered complete (*205*).
- **Minimum interval or age.** Vaccine doses administered ≤4 days before the minimum interval or age are considered valid. Because of the unique accelerated schedule for Twinrix, the 4-day guideline does not apply to the first 3 doses of this vaccine when administered on a 0-day, 7-day, 21–30-day, and 12-month schedule (*306*).

#### Other Vaccination Management Issues and Considerations

- **Dose and schedule.** HepA vaccines should only be administered with the age-appropriate dose and schedule. Administering 2 doses of 25 units of Vaqta or 2 doses of 720 ELISA units of Havrix at the same time or sequentially to persons aged ≥19 years instead of 1 adult dose is not recommended and is not included as a method for dosage and administration in the manufacturers’ package inserts; the package inserts for Havrix and Vaqta include additional information (*202*).
- **Twinrix and PEP.** Twinrix is not recommended for PEP.
- **Vaccine interchangeability.** ACIP prefers that doses of HepA vaccine in a series come from the same manufacturer; however, if this is not possible or if the manufacturer of doses given previously is unknown, providers should administer the vaccine that they have available (*205*). The dose should be considered valid and does not need to be repeated. No differences in immunogenicity have been observed when 1 valid dose of HepA vaccine produced by one manufacturer is followed by a dose from a different manufacturer, administered according to the recommended schedule (*307*,*308*); the package inserts for Vaqta include additional information (*202*).
  - Single-antigen HepA and HepB vaccines may be used in conjunction with Twinrix to form a complete series of these vaccines. Because the hepatitis B component of Twinrix is equivalent to a standard adult dose of HepB vaccine, the schedule is the same regardless of which vaccine is used for which dose. Because the hepatitis A component of Twinrix is equivalent to a pediatric dose of HepA vaccine, a series mixing the single-antigen HepA vaccine and Twinrix is more complex.
  - A person aged >19 years who receives 1 dose of Twinrix can complete the HepA vaccine series with 2 doses of adult formulation HepA vaccine separated by at least 5 months. A person who receives 2 doses of Twinrix can complete the HepA vaccine series with 1 dose of adult formulation HepA vaccine or Twinrix 5 months after the second dose. A person aged ≥19 years who begins the hepatitis A series with single-antigen HepA vaccine may complete the series with 2 doses of Twinrix or 1 dose of adult formulation HepA vaccine. Persons who are aged 18 years should follow the same schedule as for adults, using the pediatric formulations of HepA vaccine. Providers should only accept dated records as evidence of HepA vaccination. Additional information on HepB vaccination is available (*306*). (The Twinrix package insert and related references provide more information on equivalent protection following the accelerated, alternate Twinrix schedule [*202**,**283**,**309**,**310*].)
- **Susceptible persons.** Vaccination should occur among susceptible persons (i.e., unvaccinated, partially vaccinated, nonresponders to vaccine or never infected).
- **Vaccine records.** Although vaccinations should not be postponed if records cannot be found, an attempt to locate missing records should be made by contacting previous health care personnel, reviewing state or local immunization information systems, or searching for a personally held record. If records cannot be located within a reasonable time, these persons should be considered susceptible and vaccinated with the appropriate dose and schedule.
- **Vaccine series initiation.** In all settings, concern about loss to follow-up before HepA vaccine series completion should not be a deterrent to initiating the vaccine series in persons for whom HepA vaccination is recommended. Vaccination should be initiated even though completion of the series might not be ensured. One dose of HepA vaccine provides personal protection and can contribute to herd immunity, although long-term protection might be suboptimal (*244*).
- **Live vaccines and IG.** Live vaccines (e.g., MMR and varicella vaccines) should be administered at least 2 weeks before (*205*) or at least 6 months after the administration of IG (*217*); the GamaSTAN package insert includes additional information (*205*,*217*). If IG must be administered <2 weeks after the administration of MMR or varicella vaccine, the patient should be revaccinated no sooner than 6 months after receipt of IG unless serologic testing is feasible and indicates a response to that vaccine (*205*,*217*).
- **Vaccine and IG.** Persons administered IG for whom HepA vaccine is also recommended should receive a dose of vaccine simultaneously with IG in a different anatomic site (e.g., separate limbs). If only IG or only vaccine is available, either available product should be administered as soon as possible. The person may return for the other product when available, within 2 weeks of exposure if the product is administered as part of postexposure prophylaxis.
- **Standing orders.** Implementation of standing orders should be considered to overcome barriers and increase coverage.
- **Vaccine adverse events.** Adverse events occurring after administration of any vaccine should be reported to VAERS. Reports can be submitted to VAERS online, by fax, or by mail. More information about VAERS is available by telephone (800–822–7967) and online (https://vaers.hhs.gov).

## Future Directions

ACIP and CDC will review these recommendations as new epidemiology or other information related to HepA vaccines (including licensure of additional hepatitis A–containing vaccines), HepA vaccine adverse events, and the experience gained in the implementation of these recommendations becomes available. Revised recommendations will be developed as needed.

References 1. Lednar
WM, Lemon
SM,
Kirkpatrick
JW, Redfield
RR, Fields
ML, Kelley
PW. Frequency of illness
associated with epidemic hepatitis A virus infections in adults.
Am J Epidemiol
1985;122:226–33.
10.1093/oxfordjournals.aje.a11409338600022. Kemmer
NM, Miskovsky
EP. Hepatitis
A.
Infect Dis Clin North Am
2000;14:605–15.
10.1016/S0891-5520(05)70123-9109871123. Keeffe
E. Hepatitis A in patients
with chronic liver disease - severity of illness and prevention with
vaccination.
J Viral Hepat
2000;7(Suppl
1):15–7. 10.1046/j.1365-2893.2000.00016.x108701754. Rubin
LG, Levin
MJ, Ljungman
P, 
Infectious Diseases Society of America. 2013 IDSA
clinical practice guideline for vaccination of the immunocompromised
host.
Clin Infect Dis
2014;58:309–18.
10.1093/cid/cit816244213065. Glikson
M, Galun
E, Oren
R, Tur-Kaspa
R, Shouval
D. Relapsing hepatitis A.
Review of 14 cases and literature survey.
Medicine (Baltimore)
1992;71:14–23.
10.1097/00005792-199201000-0000213126596. CDC. Prevention
of hepatitis A through active or passive immunization: recommendations of the Advisory
Committee on Immunization Practices, the American Academy of Pediatrics, the American
Academy of Family Physicians, and the American Medical Association.
MMWR Recomm Rep
1996;45(No.
RR-13).7. CDC. Prevention
of hepatitis A through active or passive immunization: Recommendations of the Advisory
Committee on Immunization Practices (ACIP).
MMWR Recomm Rep
1999;48(No. RR-12).105436578. Fiore
AE, Wasley
A, Bell
BP; Advisory Committee on
Immunization Practices (ACIP). Prevention of hepatitis A
through active or passive immunization: recommendations of the Advisory Committee on
Immunization Practices (ACIP).
MMWR Recomm Rep
2006;55(No. RR-7).167080589. CDC. Surveillance for viral
hepatitis—United States, 2017. Atlanta, GA: US Department of Health and Human
Services, CDC; 2019. https://www.cdc.gov/hepatitis/statistics/SurveillanceRpts.htm10. Collier
MG, Khudyakov
YE, Selvage
D, 
Hepatitis A Outbreak Investigation Team. Outbreak of
hepatitis A in the USA associated with frozen pomegranate arils imported from Turkey:
an epidemiological case study.
Lancet Infect Dis
2014;14:976–81.
10.1016/S1473-3099(14)70883-72519517811. Viray
MA,
Hofmeister
MG, Johnston
DI,  Public health investigation and
response to a hepatitis A outbreak from imported scallops consumed raw—Hawaii,
2016. Epidemiol Infect
2018;147:1–8.10.1017/S09502688180028443032698612. Foster
M,
Ramachandran
S, Myatt
K, 
Hepatitis A virus outbreaks associated with drug use and
homelessness—California, Kentucky, Michigan, and Utah, 2017.
MMWR Morb Mortal Wkly Rep
2018;67:1208–10.
10.15585/mmwr.mm6743a330383739PMC631980113. Yin
S, Barker
L, Ly
KN, 
Susceptibility to hepatitis A virus infection in the United States,
2007–2016.
Clin Infect Dis
2020;ciaa298. 10.1093/cid/ciaa29832193542PMC1100979314. CDC. Vaccination coverage among
adults in the United States, National Health Interview Survey, 2017.
Atlanta, GA: US Department of Health and Human Services, CDC; 2018. https://www.cdc.gov/vaccines/imz-managers/coverage/adultvaxview/pubs-resources/NHIS-2017.html#box215. Doshani
M, Weng
M, Moore
KL, Romero
JR, Nelson
NP. Recommendations of the
Advisory Committee on Immunization Practices for use of hepatitis A vaccine for
persons experiencing homelessness.
MMWR Morb Mortal Wkly Rep
2019;68:153–6.
10.15585/mmwr.mm6806a630763295PMC637565316. CDC; Advisory
Committee on Immunization Practices. Updated recommendations
from the Advisory Committee on Immunization Practices (ACIP) for use of hepatitis A
vaccine in close contacts of newly arriving international adoptees.
MMWR Morb Mortal Wkly Rep
2009;58:1006–7.1976307717. Nelson
NP,
Link-Gelles
R, Hofmeister
MG, 
Update: recommendations of the Advisory Committee on Immunization
Practices for use of hepatitis A vaccine for postexposure prophylaxis and for
preexposure prophylaxis for international travel.
MMWR Morb Mortal Wkly Rep
2018;67:1216–20.
10.15585/mmwr.mm6743a530383742PMC631979818. Shimabukuro
TT, Nguyen
M, Martin
D, DeStefano
F. Safety monitoring in the
Vaccine Adverse Event Reporting System (VAERS).
Vaccine
2015;33:4398–405.
10.1016/j.vaccine.2015.07.03526209838PMC463220419. Zhou
W, Pool
V, Iskander
JK, 
Surveillance for safety after immunization: Vaccine Adverse Event
Reporting System (VAERS)—United States, 1991-2001.
MMWR Surveill Summ
2003;52(No. SS-1).1282554320. McNeil
MM, Gee
J, Weintraub
ES, 
The Vaccine Safety Datalink: successes and challenges monitoring vaccine
safety.
Vaccine
2014;32:5390–8.
10.1016/j.vaccine.2014.07.07325108215PMC672785121. Hill
HA, Singleton
JA, Yankey
D, Elam-Evans
LD, Pingali
SC, Kang
Y. Vaccination coverage by age
24 months among children born in 2015 and 2016—National Immunization
Survey—Child, United States, 2016–2018.
MMWR Morb Mortal Wkly Rep
2019;68:913–8.
10.15585/mmwr.mm6841e231622284PMC680267922. CDC. Atlanta, GA: US Department of
Health and Human Services. Widespread person-to-person outbreaks of hepatitis A across
the United States. https://www.cdc.gov/hepatitis/outbreaks/2017March-HepatitisA.htm23. Latash
J,
Dorsinville
M, Del Rosso
P, 
Notes from the field: increase in reported hepatitis A infections among
men who have sex with men—New York City, January–August
2017.
MMWR Morb Mortal Wkly Rep
2017;66:999–1000.
10.15585/mmwr.mm6637a728934181PMC565778324. National Institutes of Health, National
Institute on Drug Abuse. Substance use in women. Bethesda, MD: National
Institutes of Health, National Institute on Drug Abuse; 2018. https://www.drugabuse.gov/publications/research-reports/substance-use-in-women/summary25. Melnick
JL. Properties and
classification of hepatitis A virus.
Vaccine
1992;10(Suppl
1):S24–6. 10.1016/0264-410X(92)90536-S133565326. Martin
A, Lemon
SM. Hepatitis A virus: from
discovery to vaccines.
Hepatology
2006;43(Suppl
1):S164–72. 10.1002/hep.210521644725927. Zell
R, Delwart
E, Gorbalenya
AE, 
Ictv Report Consortium. ICTV virus taxonomy profile:
*Picornaviridae.*
J Gen Virol
2017;98:2421–2.
10.1099/jgv.0.00091128884666PMC572599128. Cohen
JI. Hepatitis A virus:
insights from molecular biology.
Hepatology
1989;9:889–95.
10.1002/hep.1840090617254106029. Lemon
SM, Ott
JJ, Van Damme
P, Shouval
D. Type A viral hepatitis: a summary and
update on the molecular virology, epidemiology, pathogenesis and prevention. J Hepatol
2017:S0168–8278(17)32278-X.10.1016/j.jhep.2017.08.0342888716430. Robertson
BH, Jansen
RW, Khanna
B, 
Genetic relatedness of hepatitis A virus strains recovered from different
geographical regions.
J Gen Virol
1992;73:1365–77.
10.1099/0022-1317-73-6-1365131894031. Costa-Mattioli
M, Napoli
AD,
Ferré
V, Billaudel
S,
Perez-Bercoff
R, Cristina
J. Genetic variability of
hepatitis A virus.
J Gen Virol
2003;84:3191–201.
10.1099/vir.0.19532-01464590132. Vaughan
G, Goncalves
Rossi
LM, Forbi
JC, 
Hepatitis A virus: host interactions, molecular epidemiology and
evolution.
Infect Genet Evol
2014;21:227–43.
10.1016/j.meegid.2013.10.0232420058733. Tassopoulos
NC,
Papaevangelou
GJ, Ticehurst
JR, Purcell
RH. Fecal excretion of Greek
strains of hepatitis A virus in patients with hepatitis A and in experimentally
infected chimpanzees.
J Infect Dis
1986;154:231–7.
10.1093/infdis/154.2.231301400934. Smith
PF, Grabau
JC,
Werzberger
A, 
The role of young children in a community-wide outbreak of hepatitis
A.
Epidemiol Infect
1997;118:243–52.
10.1017/S09502688970074629207735PMC280880635. Staes
CJ, Schlenker
TL, Risk
I, 
Sources of infection among persons with acute hepatitis A and no
identified risk factors during a sustained community-wide outbreak.
Pediatrics
2000;106:e54. 10.1542/peds.106.4.e541101554936. Fiore
AE. Hepatitis A transmitted by
food.
Clin Infect Dis
2004;38:705–15.
10.1086/3816711498625637. Barrett
CE, Pape
BJ, Benedict
KM, 
Impact of public health interventions on drinking water-associated
outbreaks of hepatitis A—United States, 1971–2017.
MMWR Morb Mortal Wkly Rep
2019;68:766–70.
10.15585/mmwr.mm6835a43148727738. McCaustland
KA, Bond
WW, Bradley
DW, Ebert
JW, Maynard
JE. Survival of hepatitis A
virus in feces after drying and storage for 1 month.
J Clin Microbiol
1982;16:957–8.
10.1128/JCM.16.5.957-958.19826296198PMC27250939. Niu
MT, Polish
LB, Robertson
BH, 
Multistate outbreak of hepatitis A associated with frozen
strawberries.
J Infect Dis
1992;166:518–24.
10.1093/infdis/166.3.518132361840. Nordic Outbreak Investigation
Team. Joint analysis by the Nordic countries of a hepatitis A
outbreak, October 2012 to June 2013: frozen strawberries suspected.
Euro Surveill
2013;18:20520. 10.2807/1560-7917.ES2013.18.27.205202387007641. Reid
TM, Robinson
HG. Frozen raspberries and
hepatitis A.
Epidemiol Infect
1987;98:109–12.
10.1017/S095026880006177X3030789PMC223528742. Favero
MSBW. Disinfection and sterilization. In:
Zuckerman AJ TH, ed. Viral hepatitis, scientific basis and clinical management. New
York: Churchill Livingstone; 1993. pp. 565–75.43. Lemon
SM. The natural history of
hepatitis A: the potential for transmission by transfusion of blood or blood
products.
Vox Sang
1994;67(Suppl
4):19–23, discussion 24–6.
10.1159/000462759783186544. Soucie
JM, Robertson
BH, Bell
BP,
McCaustland
KA, Evatt
BL. Hepatitis A virus
infections associated with clotting factor concentrate in the United
States.
Transfusion
1998;38:573–9.
10.1046/j.1537-2995.1998.38698326337.x966169145. Benjamin
RJ. Nucleic acid testing:
update and applications.
Semin Hematol
2001;38(Suppl
9):11–6. 10.1016/S0037-1963(01)90132-51168572146. Cohen
JI, Feinstone
S, Purcell
RH. Hepatitis A virus
infection in a chimpanzee: duration of viremia and detection of virus in saliva and
throat swabs.
J Infect Dis
1989;160:887–90.
10.1093/infdis/160.5.887257265347. Parry
JV, Perry
KR, Panday
S, Mortimer
PP. Diagnosis of hepatitis A
and B by testing saliva.
J Med Virol
1989;28:255–60.
10.1002/jmv.1890280410255058548. Mackiewicz
V, Dussaix
E, Le
Petitcorps
MF,
Roque-Afonso
AM. Detection of hepatitis A
virus RNA in saliva.
J Clin Microbiol
2004;42:4329–31.
10.1128/JCM.42.9.4329-4331.200415365037PMC51632449. Krugman
S, Giles
JP. Viral hepatitis. New light
on an old disease.
JAMA
1970;212:1019–29.
10.1001/jama.1970.03170190035005419150250. Lemon
SM. Type A viral hepatitis.
New developments in an old disease.
N Engl J Med
1985;313:1059–67.
10.1056/NEJM198510243131706241335651. Hadler
SC, Webster
HM, Erben
JJ, Swanson
JE, Maynard
JE. Hepatitis A in day-care
centers. A community-wide assessment.
N Engl J Med
1980;302:1222–7.
10.1056/NEJM198005293022203624536352. Tong
MJ, el-Farra
NS, Grew
MI. Clinical manifestations of
hepatitis A: recent experience in a community teaching hospital.
J Infect Dis
1995;171(Suppl
1):S15–8. 10.1093/infdis/171.Supplement_1.S15787664153. Koff
RS. Clinical manifestations
and diagnosis of hepatitis A virus infection.
Vaccine
1992;10(Suppl
1):S15–7. 10.1016/0264-410X(92)90533-P133564954. Sjogren
MH, Tanno
H, Fay
O, 
Hepatitis A virus in stool during clinical relapse.
Ann Intern Med
1987;106:221–6.
10.7326/0003-4819-106-2-221302621355. Foster
MA,
Hofmeister
MG, Kupronis
BA, 
Increase in hepatitis A virus infections—United States,
2013–2018.
MMWR Morb Mortal Wkly Rep
2019;68:413–5.
10.15585/mmwr.mm6818a231071072PMC654219156. Robertson
BH, Averhoff
F, Cromeans
TL, 
Genetic relatedness of hepatitis A virus isolates during a community-wide
outbreak.
J Med Virol
2000;62:144–50.
10.1002/1096-9071(200010)62:2<144::AID-JMV4>3.0.CO;2-I1100224257. Rosenblum
LS, Villarino
ME, Nainan
OV, 
Hepatitis A outbreak in a neonatal intensive care unit: risk factors for
transmission and evidence of prolonged viral excretion among preterm
infants.
J Infect Dis
1991;164:476–82.
10.1093/infdis/164.3.476165135958. Bower
WA, Nainan
OV, Han
X, Margolis
HS. Duration of viremia in
hepatitis A virus infection.
J Infect Dis
2000;182:12–7.
10.1086/3157011088257659. Tjon
GM, Coutinho
RA, van den
Hoek
A, 
High and persistent excretion of hepatitis A virus in immunocompetent
patients.
J Med Virol
2006;78:1398–405.
10.1002/jmv.207111699888360. Costa-Mattioli
M, Allavena
C, Poirier
AS, Billaudel
S, Raffi
F,
Ferré
V. Prolonged hepatitis A
infection in an HIV-1 seropositive patient.
J Med Virol
2002;68:7–11. 10.1002/jmv.101631221042461. Foster
MA, Weil
LM, Jin
S, 
Transmission of hepatitis A virus through combined liver-small
intestine-pancreas transplantation.
Emerg Infect Dis
2017;23:590–6.
10.3201/eid2304.16153228322704PMC536742062. Liaw
YF, Yang
CY, Chu
CM, Huang
MJ. Appearance and persistence
of hepatitis A IgM antibody in acute clinical hepatitis A observed in an
outbreak.
Infection
1986;14:156–8.
10.1007/BF01645253375924363. Lemon
SM, Murphy
PC, Provost
PJ, 
Immunoprecipitation and virus neutralization assays demonstrate
qualitative differences between protective antibody responses to inactivated hepatitis
A vaccine and passive immunization with immune globulin.
J Infect Dis
1997;176:9–19.
10.1086/514044920734464. American Medical Association;
American Nurses Association-American Nurses Foundation;
CDC; Center for Food Safety and Applied Nutrition, Food and
Drug Administration; Food Safety and Inspection Service, US
Department of Agriculture. Diagnosis and management of
foodborne illnesses: a primer for physicians and other health care
professionals.
MMWR Recomm Rep
2004;53(No. RR-4).1512398465. Kao
HW, Ashcavai
M, Redeker
AG. The persistence of
hepatitis A IgM antibody after acute clinical hepatitis A.
Hepatology
1984;4:933–6. 10.1002/hep.1840040525609029366. Sikuler
E, Keynan
A, Hanuka
N,
Zagron-Bachir
G, Sarov
I. Persistence of a positive
test for IgM antibodies to hepatitis A virus in late convalescent
sera.
Isr J Med Sci
1987;23:193–5.358370167. CDC. Positive
test results for acute hepatitis A virus infection among persons with no recent
history of acute hepatitis—United States, 2002-2004.
MMWR Morb Mortal Wkly Rep
2005;54:453–6.1588900668. Castrodale
L, Fiore
A, Schmidt
T. Detection of immunoglobulin
M antibody to hepatitis A virus in Alaska residents without other evidence of
hepatitis.
Clin Infect Dis
2005;41:e86–8.
10.1086/4970731620609269. Decker
RH, Overby
LR, Ling
CM,
Frösner
G, Deinhardt
F, Boggs
J. Serologic studies of
transmission of hepatitis A in humans.
J Infect Dis
1979;139:74–82.
10.1093/infdis/139.1.7422033070. Lemon
SM, Binn
LN. Serum neutralizing
antibody response to hepatitis A virus.
J Infect Dis
1983;148:1033–9.
10.1093/infdis/148.6.1033631776671. Locarnini
SA, Ferris
AA, Lehmann
NI, Gust
ID. The antibody response
following hepatitis A infection.
Intervirology
1977;8:309–18.
10.1159/00014890519591372. Stapleton
JT. Host immune response to
hepatitis A virus.
J Infect Dis
1995;171(Suppl
1):S9–14. 10.1093/infdis/171.Supplement_1.S9787665473. Amon
JJ, Devasia
R, Xia
G, 
Molecular epidemiology of foodborne hepatitis A outbreaks in the United
States, 2003.
J Infect Dis
2005;192:1323–30.
10.1086/4624251617074874. Nainan
OV, Armstrong
GL, Han
XH, Williams
I, Bell
BP, Margolis
HS. Hepatitis A molecular
epidemiology in the United States, 1996–1997: sources of infection and
implications of vaccination policy.
J Infect Dis
2005;191:957–63.
10.1086/4279921571727275. Hutin
YJ, Pool
V, Cramer
EH, 
National Hepatitis A Investigation Team. A multistate,
foodborne outbreak of hepatitis A.
N Engl J Med
1999;340:595–602.
10.1056/NEJM1999022534008021002964376. Nainan
OV, Xia
G, Vaughan
G, Margolis
HS. Diagnosis of hepatitis A
virus infection: a molecular approach.
Clin Microbiol Rev
2006;19:63–79.
10.1128/CMR.19.1.63-79.200616418523PMC136027177. Funkhouser
AW, Purcell
RH,
D’Hondt
E, Emerson
SU. Attenuated hepatitis A
virus: genetic determinants of adaptation to growth in MRC-5 cells.
J Virol
1994;68:148–57.
10.1128/JVI.68.1.148-157.19948254724PMC23627378. Cohen
JI, Rosenblum
B, Feinstone
SM, Ticehurst
J, Purcell
RH. Attenuation and cell
culture adaptation of hepatitis A virus (HAV): a genetic analysis with HAV
cDNA.
J Virol
1989;63:5364–70.
10.1128/JVI.63.12.5364-5370.19892555561PMC25120379. Steffen
R, Kane
MA, Shapiro
CN, Billo
N,
Schoellhorn
KJ, van Damme
P. Epidemiology and prevention
of hepatitis A in travelers.
JAMA
1994;272:885–9.
10.1001/jama.1994.03520110065031807816780. Jacobsen
KH. Globalization and the
changing epidemiology of hepatitis A virus.
Cold Spring Harb Perspect Med
2018;8:a031716. 10.1101/cshperspect.a03171629500305PMC616998681. Mohd Hanafiah
K, Jacobsen
KH, Wiersma
ST. Challenges to mapping the
health risk of hepatitis A virus infection.
Int J Health Geogr
2011;10:57. 10.1186/1476-072X-10-5722008459PMC321009082. Nelson
N. Hepatitis A. CDC yellow book 2020. New
York, NY: Oxford University Press; 2020.83. Cotter
SM, Sansom
S, Long
T, 
Outbreak of hepatitis A among men who have sex with men: implications for
hepatitis A vaccination strategies.
J Infect Dis
2003;187:1235–40.
10.1086/3740571269600284. Regan
DG, Wood
JG, Benevent
C, 
Estimating the critical immunity threshold for preventing hepatitis A
outbreaks in men who have sex with men.
Epidemiol Infect
2016;144:1528–37.
10.1017/S095026881500260526566273PMC915056985. European Centre for Disease Prevention and
Control. Hepatitis A outbreaks in the EU/EEA mostly affecting men who have
sex with men—third update. Stockholm, Sweden: European Centre for Disease
Prevention and Control; 2017. https://www.ecdc.europa.eu/en/publications-data/rapid-risk-assessment-hepatitis-outbreak-eueea-mostly-affecting-men-who-have-sex86. Ndumbi
P, Freidl
GS, Williams
CJ,  Hepatitis A outbreak
disproportionately affecting men who have sex with men (MSM) in the European Union and
European Economic Area, June 2016 to May 2017. Euro Surveill
2018;23:08.10.2807/1560-7917.ES.2018.23.33.1700641PMC62052543013109587. Plunkett
J, Mandal
S, Balogun
K,  Hepatitis A outbreak among men who
have sex with men (MSM) in England, 2016–2018: the contribution of past and
current vaccination policy and practice. Vaccine X
2019;1:100014.10.1016/j.jvacx.2019.100014PMC66682203138473688. European Centre for Disease Prevention and
Control. Epidemiological update: hepatitis A outbreak in the EU/EEA mostly
affecting men who have sex with men. Stockholm, Sweden: European Centre for Disease
Prevention and Control; 2017. https://www.ecdc.europa.eu/en/news-events/epidemiological-update-hepatitis-outbreak-eueea-mostly-affecting-men-who-have-sex-men-289. Gozlan
Y, Bar-Or
I, Rakovsky
A, 
Ongoing hepatitis A among men who have sex with men (MSM) linked to
outbreaks in Europe in Tel Aviv area, Israel, December 2016 - June
2017.
Euro Surveill
2017;22:30575. 10.2807/1560-7917.ES.2017.22.29.3057528749336PMC553296290. Rivas
V, Barrera
A, Pino
K, 
Hepatitis A outbreak since November 2016 affecting men who have sex with
men (MSM) in Chile connected to the current outbreak in MSM in Europe, situation up to
October 2017. Euro Surveill
2018;23. 10.2807/1560-7917.ES.2018.23.9.18-00060PMC58409222951078091. Tanaka
S, Kishi
T, Ishihara
A, 
Outbreak of hepatitis A linked to European outbreaks among men who have
sex with men in Osaka, Japan, from March to July 2018.
Hepatol Res
2019;49:705–10.
10.1111/hepr.133143065679392. Srivastav
A,
O’Halloran
A, Lu
PJ, Williams
WW, Hutchins
SS. Vaccination differences
among U.S. adults by their self-identified sexual orientation, National Health
Interview Survey, 2013-2015.
PLoS One
2019;14:e0213431. 10.1371/journal.pone.021343130845220PMC640520093. Whitehead
J, Shaver
J, Stephenson
R. Outness, stigma, and
primary health care utilization among rural LGBT populations.
PLoS One
2016;11:e0146139. 10.1371/journal.pone.014613926731405PMC470147194. Bialek
SR, Barry
V, Bell
BP, ; Young Men’s
Survey Study Group. Seroprevalence and correlates of hepatitis
A among HIV-negative American men who have sex with men.
Sex Health
2011;8:343–8. 10.1071/SH101622185177495. Siconolfi
DE, Halkitis
PN, Rogers
ME. Hepatitis vaccination and
infection among gay, bisexual, and other men who have sex with men who attend gyms in
New York City.
Am J Men Health
2009;3:141–9. 10.1177/15579883083151511947772796. Vong
S, Fiore
AE, Haight
DO, 
Vaccination in the county jail as a strategy to reach high risk adults
during a community-based hepatitis A outbreak among methamphetamine drug
users.
Vaccine
2005;23:1021–8.
10.1016/j.vaccine.2004.07.0381562047597. Lugoboni
F, Pajusco
B, Albiero
A, Quaglio
G. Hepatitis A virus among
drug users and the role of vaccination: a review.
Front Psychiatry
2012;2:79. 10.3389/fpsyt.2011.0007922347865PMC327633898. Hutin
YJ, Sabin
KM, Hutwagner
LC, 
Multiple modes of hepatitis A virus transmission among methamphetamine
users.
Am J Epidemiol
2000;152:186–92.
10.1093/aje/152.2.1861090995699. Collier
MG, Drobeniuc
J,
Cuevas-Mota
J, Garfein
RS, Kamili
S, Teshale
EH. Hepatitis A and B among
young persons who inject drugs—vaccination, past, and present
infection.
Vaccine
2015;33:2808–12.
10.1016/j.vaccine.2015.04.01925889161100. Koepke
R, Sill
DN, Akhtar
WZ, 
Hepatitis A and hepatitis B vaccination coverage among persons who inject
drugs and have evidence of hepatitis C infection.
Public Health Rep
2019;134:651–9.
10.1177/003335491987408831539482PMC6832086101. Hinthorn
DR, Foster
MT
Jr, Bruce
HL, Aach
RD. An outbreak of chimpanzee
associated hepatitis.
J Occup Med
1974;16:388–91.4836166102. Dienstag
JL, Davenport
FM, McCollum
RW, Hennessy
AV, Klatskin
G, Purcell
RH. Nonhuman
primate-associated viral hepatitis type A. Serologic evidence of hepatitis A virus
infection.
JAMA
1976;236:462–4.
10.1001/jama.1976.03270050018021180303103. Robertson
BH. Viral hepatitis and
primates: historical and molecular analysis of human and nonhuman primate hepatitis A,
B, and the GB-related viruses.
J Viral Hepat
2001;8:233–42.
10.1046/j.1365-2893.2001.00295.x11454173104. Advisory Committee on Immunization
Practices; CDC. Immunization of health-care
personnel: recommendations of the Advisory Committee on Immunization Practices
(ACIP).
MMWR Recomm Rep
2011;60(No. RR-7).22108587105. Raabe
VN, Sautter
C, Chesney
M, Eckerle
JK, Howard
CR, John
CC. Hepatitis A screening for
internationally adopted children from hepatitis A endemic countries.
Clin Pediatr (Phila)
2014;53:31–7. 10.1177/000992281350590324137028106. Fischer
GE, Teshale
EH, Miller
C, 
Hepatitis A among international adoptees and their
contacts.
Clin Infect Dis
2008;47:812–4.
10.1086/59119918684098107. Pelletier
AR, Mehta
PJ, Burgess
DR, 
An outbreak of hepatitis A among primary and secondary contacts of an
international adoptee.
Public Health Rep
2010;125:642–6.
10.1177/00333549101250050520873279PMC2924999108. Sweet
K, Sutherland
W, Ehresmann
K, Lynfield
R. Hepatitis A infection in
recent international adoptees and their contacts in Minnesota,
2007–2009.
Pediatrics
2011;128:e333–8.
10.1542/peds.2010-184021727107109. Vernon
JA, Payette
M, Chatterjee
A. Social welfare and
adolescent vaccination programs in the United States: the economic opportunities for a
systematic expansion.
Soc Work Public Health
2009;24:414–45.
10.1080/1937191090303816519731186110. Wilson
ME, Kimble
J. Posttravel hepatitis A:
probable acquisition from an asymptomatic adopted child.
Clin Infect Dis
2001;33:1083–5.
10.1086/32320011528585111. CDC. Viral hepatitis
surveillance—United States, 2009. Atlanta, GA: US Department of
Health and Human Services. https://www.cdc.gov/hepatitis/statistics/2009surveillance/index.htm112. National Health Care for the Homeless
Council [Internet]. Understanding homelessness: frequently asked questions.
Nashville, TN: National Health Care for the Homeless Council. https://nhchc.org/understanding-homelessness/faq/113. Health Centers, 42 U.S.C. Sect. 254b. https://www.law.cornell.edu/uscode/text/42/254b#tab_default_2114. Health Resources and Services Administration,
Bureau of Primary Health Care. Health center program compliance manual. Glossary: 330(h)
homeless population. Rockville, MD: Health Resources and Services Administration.
https://bphc.hrsa.gov/programrequirements/compliancemanual/glossary.html115. Doran
KM, Rahai
N, McCormack
RP, 
Substance use and homelessness among emergency department
patients.
Drug Alcohol Depend
2018;188:328–33.
10.1016/j.drugalcdep.2018.04.02129852450PMC6478031116. DeGroote
NP, Mattson
CL, Tie
Y, Brooks
JT, Garg
S, Weiser
J. Hepatitis A virus immunity
and vaccination among at-risk persons receiving HIV medical care.
Prev Med Rep
2018;11:139–44.
10.1016/j.pmedr.2018.06.00630003012PMC6040111117. Moon
AM, Lowy
E, Maier
MM, 
Hepatitis A virus prevention and vaccination within and outside the
Veterans Health Administration in light of recent outbreaks.
Fed Pract
2018;35(Suppl
2):S32–7.30766392PMC6375401118. Sun
HY, Kung
HC, Ho
YC, 
Seroprevalence of hepatitis A virus infection in persons with HIV
infection in Taiwan: implications for hepatitis A vaccination.
Int J Infect Dis
2009;13:e199–205.
10.1016/j.ijid.2008.12.00919208490119. Overton
ET,
Nurutdinova
D,
Sungkanuparph
S, Seyfried
W, Groger
RK, Powderly
WG. Predictors of immunity
after hepatitis A vaccination in HIV-infected persons.
J Viral Hepat
2007;14:189–93.
10.1111/j.1365-2893.2006.00822.x17305885120. Sheth
AN, Moore
RD, Gebo
KA. Provision of general and
HIV-specific health maintenance in middle aged and older patients in an urban HIV
clinic.
AIDS Patient Care STDS
2006;20:318–25.
10.1089/apc.2006.20.31816706706121. CDC. Medical monitoring project.
US Department of Health and Human Services, CDC. https://www.cdc.gov/hiv/statistics/systems/mmp/resources.html122. Adejumo
A, Sivapalan
V, Mannheimer
S. An assessment of the
seroprevalence of hepatitis A antibody and vaccination among HIV patients in an inner
city HIV clinic.
Int J Infect Dis
2010;14:e404. 10.1016/j.ijid.2010.02.518123. Koziel
MJ, Peters
MG. Viral hepatitis in HIV
infection.
N Engl J Med
2007;356:1445–54.
10.1056/NEJMra06514217409326PMC4144044124. Lin
KY, Chen
GJ, Lee
YL, 
Hepatitis A virus infection and hepatitis A vaccination in human
immunodeficiency virus-positive patients: A review.
World J Gastroenterol
2017;23:3589–606.
10.3748/wjg.v23.i20.358928611512PMC5449416125. Ciccullo
A,
Gagliardini
R, Baldin
G, 
An outbreak of acute hepatitis A among young adult men: clinical features
and HIV coinfection rate from a large teaching hospital in Rome,
Italy.
HIV Med
2018;19:369–75.
10.1111/hiv.1259729380498126. Comelli
A, Izzo
I, Casari
S, Spinetti
A, Bergamasco
A, Castelli
F. Hepatitis A outbreak in men
who have sex with men (MSM) in Brescia (Northern Italy), July 2016-July
2017.
Infez Med
2018;26:46–51.29525797127. Fonquernie
L, Meynard
JL, Charrois
A, Delamare
C, Meyohas
MC, Frottier
J. Occurrence of acute
hepatitis A in patients infected with human immunodeficiency virus.
Clin Infect Dis
2001;32:297–9.
10.1086/31847811170922128. Gallego
M, Robles
M, Palacios
R, 
Impact of acute hepatitis A virus (HAV) infection on HIV viral load in
HIV-infected patients and influence of HIV infection on acute HAV
infection.
J Int Assoc Physicians AIDS Care (Chic)
2011;10:40–2. 10.1177/154510971038569221368013129. Ida
S, Tachikawa
N, Nakajima
A, 
Influence of human immunodeficiency virus type 1 infection on acute
hepatitis A virus infection.
Clin Infect Dis
2002;34:379–85.
10.1086/33815211774086130. Laurence
JC. Hepatitis A and B
immunizations of individuals infected with human immunodeficiency
virus.
Am J Med
2005;118(Suppl
10A):75S–83S. 10.1016/j.amjmed.2005.07.02416271546131. Lee
YL, Chen
GJ, Chen
NY, 
Taiwan HIV Study Group. Less severe but prolonged course
of acute hepatitis A in human immunodeficiency virus (HIV)-infected patients compared
with HIV-uninfected patients during an outbreak: a multicenter observational
study.
Clin Infect Dis
2018;67:1595–602.
10.1093/cid/ciy32829672699132. Lombardi
A, Rossotti
R, Moioli
MC, 
The impact of HIV infection and men who have sex with men status on
hepatitis A infection: the experience of two tertiary centres in Northern Italy during
the 2017 outbreak and in the 2009–2016 period.
J Viral Hepat
2019;26:761–5.
10.1111/jvh.1308230801838133. Puoti
M, Moioli
MC, Travi
G, Rossotti
R. The burden of liver disease
in human immunodeficiency virus-infected patients.
Semin Liver Dis
2012;32:103–13.
10.1055/s-0032-131647322760649134. Yotsuyanagi
H, Koike
K, Yasuda
K, 
Prolonged fecal excretion of hepatitis A virus in adult patients with
hepatitis A as determined by polymerase chain reaction.
Hepatology
1996;24:10–3. 10.1002/hep.5102401038707246135. Mena
G,
García-Basteiro
AL,
Llupià
A, 
Factors associated with the immune response to hepatitis A vaccination in
HIV-infected patients in the era of highly active antiretroviral
therapy.
Vaccine
2013;31:3668–74.
10.1016/j.vaccine.2013.06.01223777950136. Mena
G, Vilajeliu
A, Urbiztondo
L, Bayas
JM. [Regional recommendations
on hepatitis vaccination in human immunodeficiency virus infected adult patients in
Spain: Evidence-based disparity?]
Med Clin (Barc)
2015;145:163–70.
10.1016/j.medcli.2014.12.02325771339137. André
F, Van Damme
P, Safary
A, Banatvala
J. Inactivated hepatitis A
vaccine: immunogenicity, efficacy, safety and review of official recommendations for
use.
Expert Rev Vaccines
2002;1:9–23. 10.1586/14760584.1.1.912908508138. Phung
BC, Launay
O. Vaccination against viral
hepatitis of HIV-1 infected patients.
Hum Vaccin Immunother
2012;8:554–9. 10.4161/hv.1910522634451139. Wallace
MR, Brandt
CJ, Earhart
KC, 
Safety and immunogenicity of an inactivated hepatitis A vaccine among
HIV-infected subjects.
Clin Infect Dis
2004;39:1207–13.
10.1086/42466615486846140. Dlamini
SK, Madhi
SA, Muloiwa
R, 
Guidelines for the vaccination of HIV-infected adolescents and adults in
South Africa.
South Afr J HIV Med
2018;19:8. 10.4102/sajhivmed.v19i1.839PMC1150047739449989141. Kemper
CA, Haubrich
R, Frank
I, 
California Collaborative Treatment Group. Safety and
immunogenicity of hepatitis A vaccine in human immunodeficiency virus-infected
patients: a double-blind, randomized, placebo-controlled trial.
J Infect Dis
2003;187:1327–31.
10.1086/37456212696015142. Shire
NJ, Sherman
KE. Vaccination against
hepatitis A and B in patients with HIV.
Curr Hepat Rep
2006;5:63–7. 10.1007/s11901-006-0006-z143. Crum-Cianflone
NF, Wallace
MR. Vaccination in
HIV-infected adults.
AIDS Patient Care STDS
2014;28:397–410.
10.1089/apc.2014.012125029589PMC4117268144. Van Damme
P, Banatvala
J, Fay
O, 
International Consensus Group on Hepatitis A Virus Immunity.
Hepatitis A booster vaccination: is there a need?
Lancet
2003;362:1065–71.
10.1016/S0140-6736(03)14418-214522539145. Crum-Cianflone
NF, Wilkins
K, Lee
AW, 
Infectious Disease Clinical Research Program HIV Working Group.
Long-term durability of immune responses after hepatitis A vaccination
among HIV-infected adults.
J Infect Dis
2011;203:1815–23.
10.1093/infdis/jir18021606540PMC3100512146. Huang
SH, Huang
CH, Wang
NC, 
Taiwan HIV Study Group. Early seroreversion after 2
doses of hepatitis A vaccination in human immunodeficiency virus-positive patients:
incidence and associated factors.
Hepatology
2019;70:465–75.
10.1002/hep.3049530614542PMC6767446147. Armstrong
KE, Bush
HM, Collins
JD, Feola
DJ, Caldwell
GC, Thornton
AC. Role of CD4 count in
immunity development after hepatitis A and B vaccination among HIV-infected patients:
Kentucky, 2002-2007.
J Int Assoc Physicians AIDS Care (Chic)
2010;9:179–86.
10.1177/154510971036872120530473148. Cooksley
WG. What did we learn from the
Shanghai hepatitis A epidemic?
J Viral Hepat
2000;7(Suppl
1):1–3. 10.1046/j.1365-2893.2000.00021.x10870174149. Lefilliatre
P, Villeneuve
JP. Fulminant hepatitis A in
patients with chronic liver disease.
Can J Public Health
2000;91:168–70.
10.1007/BF0340426410927841PMC6980216150. Pramoolsinsap
C. Acute hepatitis A and
acquired immunity to hepatitis A virus in hepatitis B virus (HBV) carriers and in HBV-
or hepatitis C virus-related chronic liver diseases in Thailand.
J Viral Hepat
2000;7(Suppl
1):11–2. 10.1046/j.1365-2893.2000.00017.x10866839151. Reiss
G, Keeffe
EB. Hepatitis vaccination in
patients with chronic liver disease.
Aliment Pharmacol Ther
2004;19:715–27.
10.1111/j.1365-2036.2004.01906.x15043512152. Vento
S. Fulminant hepatitis
associated with hepatitis A virus superinfection in patients with chronic hepatitis
C.
J Viral Hepat
2000;7(Suppl
1):7–8. 10.1046/j.1365-2893.2000.00019.x10866837153. Collier
MG, Tong
X, Xu
F. Hepatitis A
hospitalizations in the United States, 2002–2011.
Hepatology
2015;61:481–5.
10.1002/hep.2753725266085154. Szmuness
W, Purcell
RH, Dienstag
JL, Stevens
CE. Antibody to hepatitis A
antigen in institutionalized mentally retarded patients.
JAMA
1977;237:1702–5.
10.1001/jama.1977.03270430044016139479155. Bohm
SR, Berger
KW, Hackert
PB, . Hepatitis A
outbreak among adults with developmental disabilities in group homes—Michigan,
2013.
MMWR Morb Mortal Wkly Rep
2015;64:148–52.25695320PMC4584704156. Lim
HS, Choi
K, Lee
S. Epidemiological
investigation of an outbreak of hepatitis A at a residential facility for the
disabled, 2011.
J Prev Med Public Health
2013;46:62–73.
10.3961/jpmph.2013.46.2.6223573370PMC3615381157. Knopf-Amelung
S. Incarceration and homelessness: a
revolving door of risk. In Focus: A Quarterly Research Review of the National Health
Care for the Homeless Council 2013;2:5. https://nhchc.org/wp-content/uploads/2019/08/infocus_incarceration_nov2013.pdf158. Mumola
CJ, Karberg
JC. Drug use and dependence, state and
federal prisoners, 2004. Washington, DC: US Department of Justice, Office of Justice
Programs, Bureau of Justice Statistics; 2006.159. Skidmore
S, Parry
JV, Nottage
P. An investigation of the
potential risk of an HAV outbreak in a prison population following the introduction of
cases from a community outbreak.
Commun Dis Public Health
2001;4:133–5.11525002160. Whiteman
D, McCall
B, Falconer
A. Prevalence and determinants
of hepatitis A virus exposure among prison entrants in Queensland, Australia:
implications for public health control.
J Viral Hepat
1998;5:277–83.
10.1046/j.1365-2893.1998.00107.x9751015161. Gilbert
RL,
O’Connor
T, Mathew
S, Allen
K, Piper
M, Gill
ON. Hepatitis A
vaccination—a prison-based solution for a community-based
outbreak?
Commun Dis Public Health
2004;7:289–93.15779792162. Safran
R. Hepatitis A/B prevention at
Washington State Penitentiary.
J Investig Med
2010;58:242–3.163. Costumbrado
J, Stirland
A, Cox
G, 
Implementation of a hepatitis A/B vaccination program using an
accelerated schedule among high-risk inmates, Los Angeles County Jail,
2007–2010.
Vaccine
2012;30:6878–82.
10.1016/j.vaccine.2012.09.00622989688164. Tabor
E. The epidemiology of virus
transmission by plasma derivatives: clinical studies verifying the lack of
transmission of hepatitis B and C viruses and HIV type 1.
Transfusion
1999;39:1160–8.
10.1046/j.1537-2995.1999.39111160.x10604241165. Mannucci
PM, Gdovin
S, Gringeri
A, 
The Italian Collaborative Group. Transmission of
hepatitis A to patients with hemophilia by factor VIII concentrates treated with
organic solvent and detergent to inactivate viruses.
Ann Intern Med
1994;120:1–7. 10.7326/0003-4819-120-1-199401010-000017504424166. US Food and Drug Administration.
Guide to inspections of viral clearance processes for plasma derivatives. Silver Spring,
MD: US Food and Drug Administration; 2008. https://ntrl.ntis.gov/NTRL/dashboard/searchResults/titleDetail/PB98137144.xhtml167. Gröner
A. Pathogen safety of
plasma-derived products - Haemate P/Humate-P.
Haemophilia
2008;14(Suppl
5):54–71. 10.1111/j.1365-2516.2008.01852.x18786011PMC7165962168. Klamroth
R,
Gröner
A, Simon
TL. Pathogen inactivation and
removal methods for plasma-derived clotting factor concentrates.
Transfusion
2014;54:1406–17.
10.1111/trf.1242324117799PMC7169823169. CDC.
2016—Multistate outbreak of hepatitis A linked to frozen
strawberries (final update). Atlanta, GA: US Department of Health and Human Services,
CDC; 2016. https://www.cdc.gov/hepatitis/outbreaks/2016/hav-strawberries.htm170. Ridpath
A, Reddy
V, Layton
M, 
Hepatitis A cases among food handlers: a local health department
response—New York City, 2013.
J Public Health Manag Pract
2017;23:571–6.
10.1097/PHH.000000000000052628166179PMC6951794171. Sharapov
UM,
Kentenyants
K, Groeger
J, Roberts
H, Holmberg
SD, Collier
MG. Hepatitis A infections
among food handlers in the United States, 1993–2011.
Public Health Rep
2016;131:26–9.
10.1177/00333549161310010726843666PMC4716468172. Morey
RJ, Collier
MG, Nelson
NP. The financial burden of
public health responses to hepatitis A cases among food handlers,
2012–2014.
Public Health Rep
2017;132:443–7.
10.1177/003335491771094728609202PMC5507430173. Hofmeister
MG, Foster
MA,
Montgomery
MP, Gupta
N. Notes from the field:
assessing the role of food handlers in hepatitis A virus transmission—multiple
states, 2016–2019.
MMWR Morb Mortal Wkly Rep
2020;69:636–7.
10.15585/mmwr.mm6920a432437339174. Hadler
SC, Erben
JJ, Francis
DP, Webster
HM, Maynard
JE. Risk factors for hepatitis
A in day-care centers.
J Infect Dis
1982;145:255–61.
10.1093/infdis/145.2.2557054328175. Kuhar
DT, Carrico
RM, Cox
K,  Infection control in healthcare
personnel: infrastructure and routine practices for occupational infection prevention
and control services. Atlanta, GA: US Department of Health and Human Services, CDC;
2019. https://www.cdc.gov/infectioncontrol/pdf/guidelines/infection-control-HCP-H.pdf176. Klein
BS, Michaels
JA, Rytel
MW, Berg
KG, Davis
JP. Nosocomial hepatitis A. A
multinursery outbreak in Wisconsin.
JAMA
1984;252:2716–21.
10.1001/jama.1984.033501900180126492350177. Noble
RC, Kane
MA, Reeves
SA, Roeckel
I. Posttransfusion hepatitis A
in a neonatal intensive care unit.
JAMA
1984;252:2711–5.
10.1001/jama.1984.033501900130116492349178. Doebbeling
BN, Li
N, Wenzel
RP. An outbreak of hepatitis A
among health care workers: risk factors for transmission.
Am J Public Health
1993;83:1679–84.
10.2105/AJPH.83.12.16798259794PMC1694909179. Goodman
RA. Nosocomial hepatitis
A.
Ann Intern Med
1985;103:452–4.
10.7326/0003-4819-103-3-4524026088180. Papaevangelou
GJ,
Roumeliotou-Karayannis
AJ,
Contoyannis
PC. The risk of nosocomial
hepatitis A and B virus infections from patients under care without isolation
precaution.
J Med Virol
1981;7:143–8. 10.1002/jmv.18900702086267188181. Siegel
JD, Rhinehart
E, Jackson
M, Chiarello
L; Health Care Infection Control
Practices Advisory Committee. 2007 Guideline for isolation
precautions: preventing transmission of infectious agents in healthcare
settings.
Am J Infect Control
2007;35(Suppl
2):S65–164. 10.1016/j.ajic.2007.10.00718068815PMC7119119182. Glas
C, Hotz
P, Steffen
R. Hepatitis A in workers
exposed to sewage: a systematic review.
Occup Environ Med
2001;58:762–8.
10.1136/oem.58.12.76211706141PMC1740082183. Lerman
Y, Chodik
G, Aloni
H, Ribak
J, Ashkenazi
S. Occupations at increased
risk of hepatitis A: a 2-year nationwide historical prospective study.
Am J Epidemiol
1999;150:312–20.
10.1093/oxfordjournals.aje.a01000410430237184. Poole
CJ,
Shakespeare
AT. Should sewage workers and
carers for people with learning disabilities be vaccinated for hepatitis
A?
BMJ
1993;306:1102. 10.1136/bmj.306.6885.11028388287PMC1677505185. Trout
D, Mueller
C, Venczel
L, Krake
A. Evaluation of occupational
transmission of hepatitis A virus among wastewater workers.
J Occup Environ Med
2000;42:83–7. 10.1097/00043764-200001000-0002010652693186. Venczel
L, Brown
S, Frumkin
H,
Simmonds-Diaz
J, Deitchman
S, Bell
BP. Prevalence of hepatitis A
virus infection among sewage workers in Georgia.
Am J Ind Med
2003;43:172–8.
10.1002/ajim.1017412541272187. Weldon
M, VanEgdom
MJ, Hendricks
KA, Regner
G, Bell
BP, Sehulster
LM. Prevalence of antibody to
hepatitis A virus in drinking water workers and wastewater workers in Texas from 1996
to 1997.
J Occup Environ Med
2000;42:821–6.
10.1097/00043764-200008000-0001110953820188. CDC. Interim immunization
recommendations for individuals displaced by a disaster. Atlanta, GA: US Department of
Health and Human Services, CDC. https://www.cdc.gov/disasters/disease/vaccrecdisplaced.html189. Bloch
AB, Stramer
SL, Smith
JD, 
Recovery of hepatitis A virus from a water supply responsible for a
common source outbreak of hepatitis A.
Am J Public Health
1990;80:428–30.
10.2105/AJPH.80.4.4282156462PMC1404566190. Bowen
GS, McCarthy
MA. Hepatitis A associated
with a hardware store water fountain and a contaminated well in Lancaster County,
Pennsylvania, 1980.
Am J Epidemiol
1983;117:695–705.685902510.1093/oxfordjournals.aje.a113603191. Bergeisen
GH, Hinds
MW, Skaggs
JW. A waterborne outbreak of
hepatitis A in Meade County, Kentucky.
Am J Public Health
1985;75:161–4
10.2105/ajph.75.2.161 .396662210.2105/ajph.75.2.161PMC1645986192. Mahoney
FJ, Farley
TA, Kelso
KY, Wilson
SA, Horan
JM, McFarland
LM. An outbreak of hepatitis A
associated with swimming in a public pool.
J Infect Dis
1992;165:613–8.
10.1093/infdis/165.4.6131552192193. De Serres
G, Cromeans
TL, Levesque
B, 
Molecular confirmation of hepatitis A virus from well water: epidemiology
and public health implications.
J Infect Dis
1999;179:37–43.
10.1086/3145659841820194. Friedman
LS,
O’Brien
TF, Morse
LJ, 
Revisiting the Holy Cross football team hepatitis outbreak (1969) by
serological analysis.
JAMA
1985;254:774–6.
10.1001/jama.1985.033600600760292989569195. Bell
BP, Shapiro
CN, Alter
MJ, 
The diverse patterns of hepatitis A epidemiology in the United
States—implications for vaccination strategies.
J Infect Dis
1998;178:1579–84.
10.1086/3145189815207196. Shaw
FE
Jr, Sudman
JH, Smith
SM, 
A community-wide epidemic of hepatitis A in Ohio.
Am J Epidemiol
1986;123:1057–65.
10.1093/oxfordjournals.aje.a1143343706276197. Dentinger
CM, Bower
WA, Nainan
OV, 
An outbreak of hepatitis A associated with green onions.
J Infect Dis
2001;183:1273–6.
10.1086/31968811262211198. Rosenblum
LS, Mirkin
IR, Allen
DT, Safford
S, Hadler
SC. A multifocal outbreak of
hepatitis A traced to commercially distributed lettuce.
Am J Public Health
1990;80:1075–9.
10.2105/AJPH.80.9.10752382744PMC1404849199. CDC. Hepatitis
A outbreak associated with green onions at a restaurant—Monaca, Pennsylvania,
2003.
MMWR Morb Mortal Wkly Rep
2003;52:1155–7.14647018200. Wheeler
C, Vogt
TM, Armstrong
GL, 
An outbreak of hepatitis A associated with green onions.
N Engl J Med
2005;353:890–7.
10.1056/NEJMoa05085516135833201. Desenclos
JC, Klontz
KC, Wilder
MH, Nainan
OV, Margolis
HS, Gunn
RA. A multistate outbreak of
hepatitis A caused by the consumption of raw oysters.
Am J Public Health
1991;81:1268–72.
10.2105/AJPH.81.10.12681928524PMC1405303202. US Food and Drug Administration.
Vaccines licensed for use in the United States. Silver Spring, MD: US Food and Drug
Administration. https://www.fda.gov/vaccines-blood-biologics/vaccines/vaccines-licensed-use-united-states203. Armstrong
ME, Giesa
PA, Davide
JP, 
Development of the formalin-inactivated hepatitis A vaccine, VAQTA from
the live attenuated virus strain CR326F.
J Hepatol
1993;18(Suppl
2):S20–6. 10.1016/S0168-8278(05)80373-38182268204. Peetermans
J. Production, quality control
and characterization of an inactivated hepatitis A vaccine.
Vaccine
1992;10(Suppl
1):S99–101. 10.1016/0264-410X(92)90557-Z1335671205. Ezeanolue
E, Harriman
K, Hunter
P, Kroger
A, Pellegrini
C. General best practice guidelines for
immunization: best practices guidance of the Advisory Committee on Immunization
Practices (ACIP). Atlanta, GA: US Department of Health and Human Services, CDC;
2018. https://www.cdc.gov/vaccines/hcp/acip-recs/general-recs/index.html206. Ragni
MV, Lusher
JM, Koerper
MA,
Manco-Johnson
M, Krause
DS. Safety and immunogenicity
of subcutaneous hepatitis A vaccine in children with haemophilia.
Haemophilia
2000;6:98–103.
10.1046/j.1365-2516.2000.00386.x10781196207. Linglöf
T, van Hattum
J, Kaplan
KM, 
An open study of subcutaneous administration of inactivated hepatitis A
vaccine (VAQTA) in adults: safety, tolerability, and immunogenicity.
Vaccine
2001;19:3968–71.
10.1016/S0264-410X(01)00134-711427272208. Czeschinski
PA, Binding
N, Witting
U. Hepatitis A and hepatitis B
vaccinations: immunogenicity of combined vaccine and of simultaneously or separately
applied single vaccines.
Vaccine
2000;18:1074–80.
10.1016/S0264-410X(99)00354-010590328209. Knöll
A,
Hottenträger
B, Kainz
J,
Bretschneider
B, Jilg
W. Immunogenicity of a
combined hepatitis A and B vaccine in healthy young adults.
Vaccine
2000;18:2029–32.
10.1016/S0264-410X(99)00524-110706965210. Plotkin
SA. Correlates of protection
induced by vaccination.
Clin Vaccine Immunol
2010;17:1055–65.
10.1128/CVI.00131-1020463105PMC2897268211. Nalin
D, Brown
L, Kuter
B, 
Inactivated hepatitis A vaccine in childhood: implications for disease
control.
Vaccine
1993;11(Suppl
1):S15–7. 10.1016/0264-410X(93)90152-N8383388212. Stapleton
JT, Jansen
R, Lemon
SM. Neutralizing antibody to
hepatitis A virus in immune serum globulin and in the sera of human recipients of
immune serum globulin.
Gastroenterology
1985;89:637–42.
10.1016/0016-5085(85)90462-72991071213. Winokur
PL, Stapleton
JT. Immunoglobulin prophylaxis
for hepatitis A.
Clin Infect Dis
1992;14:580–6.
10.1093/clinids/14.2.5801554845214. Lemon
SM. Immunologic approaches to
assessing the response to inactivated hepatitis A vaccine.
J Hepatol
1993;18(Suppl
2):S15–9. 10.1016/S0168-8278(05)80372-18182266215. Clemens
R, Safary
A, Hepburn
A, Roche
C, Stanbury
WJ,
André
FE. Clinical experience with
an inactivated hepatitis A vaccine.
J Infect Dis
1995;171(Suppl
1):S44–9. 10.1093/infdis/171.Supplement_1.S447876648216. Shouval
D, Ashur
Y, Adler
R, 
Single and booster dose responses to an inactivated hepatitis A virus
vaccine: comparison with immune serum globulin prophylaxis.
Vaccine
1993;11(Suppl
1):S9–14. 10.1016/0264-410X(93)90151-M8383390217. Grifols. Treating with GamaSTAN (immune globulin
[human]). Los Angeles, CA: Grifols, 2019. https://www.hypermunes.com/en/hcp/gamastan-hepatitis-a218. Nelson
NP. Updated dosing
instructions for immune globulin (Human) GamaSTAN S/D for hepatitis A virus
prophylaxis.
MMWR Morb Mortal Wkly Rep
2017;66:959–60.
10.15585/mmwr.mm6636a528910270PMC5657912219. Tejada-Strop
A, Costafreda
MI, Dimitrova
Z, Kaplan
GG, Teo
CG. Evaluation of potencies of
immune globulin products against hepatitis A.
JAMA Intern Med
2017;177:430–2.
10.1001/jamainternmed.2016.905728097299220. Bell
BP, Negus
S, Fiore
AE, 
Immunogenicity of an inactivated hepatitis A vaccine in infants and young
children.
Pediatr Infect Dis J
2007;26:116–22.
10.1097/01.inf.0000253253.85640.cc17259872221. Dagan
R, Amir
J, Mijalovsky
A, 
Immunization against hepatitis A in the first year of life: priming
despite the presence of maternal antibody.
Pediatr Infect Dis J
2000;19:1045–52.
10.1097/00006454-200011000-0000411099084222. Letson
GW, Margolis
HS. Hepatitis A vaccination in
infants: the ultimate solution to a long-standing public health
problem.
Curr Pediatr Rev
2007;3:11–9. 10.2174/157339607779941589223. Lieberman
J, Marcy
S, Partridge
S, Ward
J, editors. Hepatitis A vaccine in infants:
effect of maternal antibodies on the antibody response [Program and abstracts].
Presented at the 36th annual Infectious Diseases Society of America meeting. Alexandria,
VA: Infectious Diseases Society of America; 1998.224. Piazza
M, Safary
A, Vegnente
A, 
Safety and immunogenicity of hepatitis A vaccine in infants: a candidate
for inclusion in the childhood vaccination programme.
Vaccine
1999;17:585–8.
10.1016/S0264-410X(98)00237-010075165225. Sharapov
UM, Bulkow
LR, Negus
SE, 
Persistence of hepatitis A vaccine induced seropositivity in infants and
young children by maternal antibody status: 10-year follow-up.
Hepatology
2012;56:516–22.
10.1002/hep.2568722371069226. Troisi
CL, Hollinger
FB, Krause
DS, Pickering
LK. Immunization of
seronegative infants with hepatitis A vaccine (HAVRIX; SKB): a comparative study of
two dosing schedules.
Vaccine
1997;15:1613–7.
10.1016/S0264-410X(97)00199-09364691227. Balcarek
KB, Bagley
MR, Pass
RF, Schiff
ER, Krause
DS. Safety and immunogenicity
of an inactivated hepatitis A vaccine in preschool children.
J Infect Dis
1995;171(Suppl
1):S70–2. 10.1093/infdis/171.Supplement_1.S707876652228. Horng
YC, Chang
MH, Lee
CY, Safary
A, Andre
FE, Chen
DS. Safety and immunogenicity
of hepatitis A vaccine in healthy children.
Pediatr Infect Dis J
1993;12:359–62.
10.1097/00006454-199305000-000018392163229. McMahon
BJ, Williams
J, Bulkow
L, 
Immunogenicity of an inactivated hepatitis A vaccine in Alaska Native
children and Native and non-Native adults.
J Infect Dis
1995;171:676–9.
10.1093/infdis/171.3.6767876615230. Nalin
D. VAQTA™: hepatitis A
vaccine, purified inactivated.
Drugs Future
1995;20:24–9. 10.1358/dof.1995.020.01.279550231. Van Herck
K, Van Damme
P. Prevention of hepatitis A
by Havrix: a review.
Expert Rev Vaccines
2005;4:459–71.
10.1586/14760584.4.4.45916117704232. Ashur
Y, Adler
R, Rowe
M, Shouval
D. Comparison of
immunogenicity of two hepatitis A vaccines—VAQTA and HAVRIX—in young
adults.
Vaccine
1999;17:2290–6.
10.1016/S0264-410X(98)00480-010403597233. Findor
JA, Cañero
Velasco
MC, Mutti
J, Safary
A. Response to hepatitis A
vaccine in children after a single dose with a booster administration 6 months
later.
J Travel Med
1996;3:156–9. 10.1111/j.1708-8305.1996.tb00730.x9815444234. Van Der Meeren
O, Crasta
P, de Ridder
M. A retrospective pooled
analysis assessing the effect of age on the immunogenicity of Havrix™ in
healthy adults.
Hum Vaccin Immunother
2015;11:1729–34.
10.1080/21645515.2015.104516726029816PMC4514304235. Briem
H, Safary
A. Immunogenicity and safety
in adults of hepatitis A virus vaccine administered as a single dose with a booster 6
months later.
J Med Virol
1994;44:443–5.
10.1002/jmv.18904404247897378236. Innis
BL, Snitbhan
R, Kunasol
P, 
Protection against hepatitis A by an inactivated vaccine.
JAMA
1994;271:1328–34.
10.1001/jama.1994.035104100400308158817237. Werzberger
A, Mensch
B, Kuter
B, 
A controlled trial of a formalin-inactivated hepatitis A vaccine in
healthy children.
N Engl J Med
1992;327:453–7.
10.1056/NEJM1992081332707021320740238. Spradling
PR, Bulkow
LR, Negus
SE, Homan
C, Bruce
MG, McMahon
BJ. Persistence of
seropositivity among persons vaccinated for hepatitis A during infancy by maternal
antibody status: 15-year follow-up.
Hepatology
2016;63:703–11.
10.1002/hep.2837526637987PMC6459008239. Plumb
ID, Bulkow
LR, Bruce
MG, 
Persistence of antibody to hepatitis A virus 20 years after
receipt of Hepatitis A vaccine in Alaska.
J Viral Hepat
2017;24:608–12.
10.1111/jvh.1267628092416240. Mosites
E, Gounder
P, Snowball
M, 
Hepatitis A vaccine immune response 22 years after
vaccination.
J Med Virol
2018;90:1418–22.
10.1002/jmv.2519729663458241. Raczniak
GA, Thomas
TK, Bulkow
LR, 
Duration of protection against hepatitis A for the current two-dose
vaccine compared to a three-dose vaccine schedule in children.
Vaccine
2013;31:2152–5.
10.1016/j.vaccine.2013.02.04823470239PMC4648811242. Hens
N, Habteab
Ghebretinsae
A, Hardt
K, Van Damme
P, Van Herck
K. Model based estimates of
long-term persistence of inactivated hepatitis A vaccine-induced antibodies in
adults.
Vaccine
2014;32:1507–13.
10.1016/j.vaccine.2013.10.08824508042243. Theeten
H, Van Herck
K, Van Der
Meeren
O, Crasta
P, Van Damme
P, Hens
N. Long-term antibody
persistence after vaccination with a 2-dose Havrix (inactivated hepatitis A vaccine):
20 years of observed data, and long-term model-based predictions.
Vaccine
2015;33:5723–7.
10.1016/j.vaccine.2015.07.00826190091244. Ott
JJ, Wiersma
ST. Single-dose administration
of inactivated hepatitis A vaccination in the context of hepatitis A vaccine
recommendations.
Int J Infect Dis
2013;17:e939–44.
10.1016/j.ijid.2013.04.01223791857245. Melgaço
JG, Morgado
LN, Santiago
MA, 
A single dose of inactivated hepatitis A vaccine promotes HAV-specific
memory cellular response similar to that induced by a natural
infection.
Vaccine
2015;33:3813–20.
10.1016/j.vaccine.2015.06.09926144899246. McMahon
BJ, Beller
M, Williams
J, Schloss
M, Tanttila
H, Bulkow
L. A program to control an
outbreak of hepatitis A in Alaska by using an inactivated hepatitis A
vaccine.
Arch Pediatr Adolesc Med
1996;150:733–9.
10.1001/archpedi.1996.021703200790148673200247. Brito
WI,
Alves-Junior
ER, Oliveira
RM, Souto
FJD. Initial evaluation of
universal immunization with a single dose against hepatitis A virus in Central
Brazil.
Braz J Infect Dis
2018;22:166–70.
10.1016/j.bjid.2018.04.00129684320PMC9425659248. Souto
FJD, de Brito
WI, Fontes
CJF. Impact of the single-dose
universal mass vaccination strategy against hepatitis A in Brazil.
Vaccine
2019;37:771–5.
10.1016/j.vaccine.2018.12.05430639462249. Urueña
A,
González
JE, Rearte
A, 
Single-dose universal hepatitis A immunization in one-year-old children
in Argentina: high prevalence of protective antibodies up to 9 years after
vaccination.
Pediatr Infect Dis J
2016;35:1339–42.
10.1097/INF.000000000000132227636725250. Urueña
A,
González
J, Carrega
MEP, 
Single-dose universal hepatitis A immunization in 1-year-old infants in
Argentina: high prevalence of protective antibodies up to 11 years following
vaccination.
Open Forum Infect Dis
2017;4(Suppl
1):S320. 10.1093/ofid/ofx163.753251. Letson
GW, Shapiro
CN, Kuehn
D, 
Effect of maternal antibody on immunogenicity of hepatitis A vaccine in
infants.
J Pediatr
2004;144:327–32.
10.1016/j.jpeds.2003.11.03015001936252. Fiore
AE, Shapiro
CN, Sabin
K, 
Hepatitis A vaccination of infants: effect of maternal antibody status on
antibody persistence and response to a booster dose.
Pediatr Infect Dis J
2003;22:354–9.
10.1097/01.inf.0000059446.52063.b912690277253. Kanra
G, Yalcin
SS, Kara
A, Ozmert
E,
Yurdakök
K. Hepatitis A booster vaccine
in children after infant immunization.
Pediatr Infect Dis J
2002;21:727–30.
10.1097/00006454-200208000-0000612192159254. Lieberman
JM, Chang
SJ, Partridge
S, 
Kinetics of maternal hepatitis A antibody decay in infants: implications
for vaccine use.
Pediatr Infect Dis J
2002;21:347–8.
10.1097/00006454-200204000-0001712075769255. Wagner
G, Lavanchy
D, Darioli
R, 
Simultaneous active and passive immunization against hepatitis A studied
in a population of travellers.
Vaccine
1993;11:1027–32.
10.1016/0264-410X(93)90128-K8212822256. Walter
EB, Hornick
RB, Poland
GA, 
Concurrent administration of inactivated hepatitis A vaccine with immune
globulin in healthy adults.
Vaccine
1999;17:1468–73.
10.1016/S0264-410X(98)00370-310195783257. Fritzsche
C, Bergmann
L, Loebermann
M, Glass
A, Reisinger
EC. Immune response to
hepatitis A vaccine in patients with HIV.
Vaccine
2019;37:2278–83.
10.1016/j.vaccine.2019.02.06430890384258. Garcia Garrido
HM, Wieten
RW, Grobusch
MP, Goorhuis
A. Response to hepatitis A
vaccination in immunocompromised travelers.
J Infect Dis
2015;212:378–85.
10.1093/infdis/jiv06025649170259. Garcia Garrido
HM, Veurink
AM, Leeflang
M, Spijker
R, Goorhuis
A, Grobusch
MP. Hepatitis A vaccine
immunogenicity in patients using immunosuppressive drugs: a systematic review and
meta-analysis.
Travel Med Infect Dis
2019;32:101479. 10.1016/j.tmaid.2019.10147931521804260. Mena
G,
García-Basteiro
AL, Bayas
JM. Hepatitis B and A
vaccination in HIV-infected adults: A review.
Hum Vaccin Immunother
2015;11:2582–98.
10.1080/21645515.2015.105542426208678PMC4685678261. Weinberg
A, Gona
P, Nachman
SA, 
Pediatric AIDS Clinical Trials Group 1008 Team. Antibody
responses to hepatitis A virus vaccine in HIV-infected children with evidence of
immunologic reconstitution while receiving highly active antiretroviral
therapy.
J Infect Dis
2006;193:302–11.
10.1086/49897916362896262. Neilsen
GA, Bodsworth
NJ, Watts
N. Response to hepatitis A
vaccination in human immunodeficiency virus-infected and -uninfected homosexual
men.
J Infect Dis
1997;176:1064–7.
10.1086/5165129333168263. Rimland
D, Guest
JL. Response to hepatitis A
vaccine in HIV patients in the HAART era.
AIDS
2005;19:1702–4.
10.1097/01.aids.0000186815.99993.5516184045264. Gouvea
AF, De
Moraes-Pinto
MI, Ono
E, 
Immunogenicity and tolerability of hepatitis A vaccine in HIV-infected
children.
Clin Infect Dis
2005;41:544–8.
10.1086/43205516028166265. Arslan
M, Wiesner
RH, Poterucha
JJ, Zein
NN. Safety and efficacy of
hepatitis A vaccination in liver transplantation recipients.
Transplantation
2001;72:272–6.
10.1097/00007890-200107270-0001911477352266. Dumot
JA, Barnes
DS, Younossi
Z, 
Immunogenicity of hepatitis A vaccine in decompensated liver
disease.
Am J Gastroenterol
1999;94:1601–4.
10.1111/j.1572-0241.1999.01150.x10364031267. Ferreira
CT, da
Silveira
TR, Vieira
SM, Taniguchi
A,
Pereira-Lima
J. Immunogenicity and safety
of hepatitis A vaccine in children with chronic liver disease.
J Pediatr Gastroenterol Nutr
2003;37:258–61.
10.1097/00005176-200309000-0001112960646268. Keeffe
EB, Iwarson
S, McMahon
BJ, 
Safety and immunogenicity of hepatitis A vaccine in patients with chronic
liver disease.
Hepatology
1998;27:881–6.
10.1002/hep.5102703369500723269. Lee
SD, Chan
CY, Yu
MI, 
Safety and immunogenicity of inactivated hepatitis A vaccine in patients
with chronic liver disease.
J Med Virol
1997;52:215–8.
10.1002/(SICI)1096-9071(199706)52:2<215::AID-JMV16>3.0.CO;2-J9179771270. Majda-Stanislawska
E, Bednarek
M, Kuydowicz
J. Immunogenicity of
inactivated hepatitis A vaccine in children with chronic liver
disease.
Pediatr Infect Dis J
2004;23:571–4.
10.1097/01.inf.0000130076.33497.6c15194843271. Stark
K,
Günther
M, Neuhaus
R, 
Immunogenicity and safety of hepatitis A vaccine in liver and renal
transplant recipients.
J Infect Dis
1999;180:2014–7.
10.1086/31512510558960272. Günther
M, Stark
K, Neuhaus
R, Reinke
P,
Schröder
K, Bienzle
U. Rapid decline of antibodies
after hepatitis A immunization in liver and renal transplant
recipients.
Transplantation
2001;71:477–9.
10.1097/00007890-200102150-0002311233913273. Reuman
PD, Kubilis
P, Hurni
W, Brown
L, Nalin
D. The effect of age and
weight on the response to formalin inactivated, alum-adjuvanted hepatitis A vaccine in
healthy adults.
Vaccine
1997;15:1157–61.
10.1016/S0264-410X(96)00310-69269062274. Tong
MJ, Co
RL, Bellak
C. Hepatitis A
vaccination.
West J Med
1993;158:602–5.8393253PMC1311784275. Link-Gelles
R, Hofmeister
MG, Nelson
NP. Use of hepatitis A vaccine
for post-exposure prophylaxis in individuals over 40 years of age: A systematic
review of published studies and recommendations for vaccine use.
Vaccine
2018;36:2745–50.
10.1016/j.vaccine.2018.04.01529673941276. Postmarketing reporting of adverse experiences. 21
CFR Sect. 600.80 (2010). https://www.accessdata.fda.gov/scripts/cdrh/cfdocs/cfcfr/CFRSearch.cfm?fr=600.80277. Moro
PL, Museru
OI, Niu
M, Lewis
P, Broder
K. Reports to the Vaccine Adverse Event
Reporting System after hepatitis A and hepatitis AB vaccines in pregnant women. Am J
Obstet Gynecol 2014;210:561:e1–6.10.1016/j.ajog.2013.12.036PMC650045024378675278. Groom
HC, Smith
N, Irving
SA, 
Uptake and safety of hepatitis A vaccination during pregnancy: a Vaccine
Safety Datalink study.
Vaccine
2019;37:6648–55.
10.1016/j.vaccine.2019.09.04331548013PMC8082525279. Bienzle
U, Bock
HL,
Kruppenbacher
JP, Hofmann
F, Vogel
GE, Clemens
R. Immunogenicity of an
inactivated hepatitis A vaccine administered according to two different schedules and
the interference of other “travellers” vaccines with the immune
response.
Vaccine
1996;14:501–5.
10.1016/0264-410X(95)00224-O8782347280. Gil
A,
González
A,
Dal-Ré
R, Calero
JR. Interference assessment of
yellow fever vaccine with the immune response to a single-dose inactivated hepatitis A
vaccine (1440 EL.U.). A controlled study in adults.
Vaccine
1996;14:1028–30.
10.1016/0264-410X(96)00059-X8879097281. Jong
EC, Kaplan
KM, Eves
KA, Taddeo
CA, Lakkis
HD, Kuter
BJ. An open randomized study
of inactivated hepatitis A vaccine administered concomitantly with typhoid fever and
yellow fever vaccines.
J Travel Med
2002;9:66–70. 10.2310/7060.2002.2195512044272282. Ambrosch
F,
André
FE, Delem
A, 
Simultaneous vaccination against hepatitis A and B: results of a
controlled study.
Vaccine
1992;10(Suppl
1):S142–5. 10.1016/0264-410X(92)90570-A1335647283. Joines
RW, Blatter
M, Abraham
B, 
A prospective, randomized, comparative US trial of a combination
hepatitis A and B vaccine (Twinrix) with corresponding monovalent vaccines (Havrix and
Engerix-B) in adults.
Vaccine
2001;19:4710–9.
10.1016/S0264-410X(01)00240-711535321284. Usonis
V, Meriste
S, Bakasenas
V, 
Immunogenicity and safety of a combined hepatitis A and B vaccine
administered concomitantly with either a measles-mumps-rubella or a
diphtheria-tetanus-acellular pertussis-inactivated poliomyelitis vaccine mixed with a
*Haemophilus influenzae* type b conjugate vaccine in infants aged
12–18 months.
Vaccine
2005;23:2602–6.
10.1016/j.vaccine.2004.11.06215780442285. Petrecz
M, Acosta
CJ, Klopfer
SO, 
Safety and immunogenicity of VAQTA® in children 12-to-23 months of
age with and without administration of other US pediatric vaccines.
Hum Vaccin Immunother
2019;15:426–32.
10.1080/21645515.2018.153093430431383PMC6422454286. Rein
DB, Hicks
KA, Wirth
KE, 
Cost-effectiveness of routine childhood vaccination for hepatitis A in
the United States.
Pediatrics
2007;119:e12–21.
10.1542/peds.2006-157317200237287. Jacobs
RJ, Meyerhoff
AS. Comparative cost
effectiveness of varicella, hepatitis A, and pneumococcal conjugate
vaccines.
Prev Med
2001;33:639–45.
10.1006/pmed.2001.093811716661288. Hankin-Wei
A, Rein
DB,
Hernandez-Romieu
A, 
Cost-effectiveness analysis of catch-up hepatitis A vaccination among
unvaccinated/partially-vaccinated children.
Vaccine
2016;34:4243–9.
10.1016/j.vaccine.2016.06.04027317459PMC5582969289. Williams
WW, Lu
PJ,
O’Halloran
A, 
Surveillance of vaccination coverage among adult
populations—United States, 2015.
MMWR Surveill Summ
2017;66. 10.15585/mmwr.ss6611a128472027PMC5829683290. McLean
HQ,
Fiebelkorn
AP, Temte
JL, Wallace
GS. Prevention of measles,
rubella, congenital rubella syndrome, and mumps, 2013: summary recommendations of the
Advisory Committee on Immunization Practices (ACIP).
MMWR Recomm Rep
2013;62(No. RR-4).23760231291. Buxton
JA, Kim
JH. Hepatitis A and hepatitis
B vaccination responses in persons with chronic hepatitis C infections: A review of
the evidence and current recommendations.
Can J Infect Dis Med Microbiol
2008;19:197–202.
10.1155/2008/41036219352452PMC2605862292. Panel on Opportunistic Infections in Adults and
Adolescents with HIV. Guidelines for the prevention and treatment of opportunistic
infections in adults and adolescents with HIV: recommendations from the Centers for
Disease Control and Prevention, the National Institutes of Health, and the HIV Medicine
Association of the Infectious Diseases Society of America. Washington, DC: US Department
of Health and Human Services. https://aidsinfo.nih.gov/contentfiles/lvguidelines/adult_oi.pdf293. Tseng
YT, Chang
SY, Liu
WC, 
Comparative effectiveness of two doses versus three doses of hepatitis A
vaccine in human immunodeficiency virus-infected and -uninfected men who have sex with
men.
Hepatology
2013;57:1734–41.
10.1002/hep.2621023258666294. Weissman
S, Feucht
C, Moore
BA. Response to hepatitis A
vaccine in HIV-positive patients.
J Viral Hepat
2006;13:81–6. 10.1111/j.1365-2893.2005.00658.x16436125295. Brennan
J, Moore
K, Sizemore
L, 
Notes from the field: acute hepatitis A virus infection among previously
vaccinated persons with HIV infection—Tennessee, 2018.
MMWR Morb Mortal Wkly Rep
2019;68:328–9.
10.15585/mmwr.mm6814a330973852PMC6459582296. Koff
RS. Hepatitis
A.
Lancet
1998;351:1643–9.
10.1016/S0140-6736(98)01304-X9620732297. Victor
JC, Surdina
TY,
Suleimeova
SZ, Favorov
MO, Bell
BP, Monto
AS. The increasing prominence
of household transmission of hepatitis A in an area undergoing a shift in
endemicity.
Epidemiol Infect
2006;134:492–7.
10.1017/S095026880500519416194291PMC2870411298. Smith
D, Huynh
C, Moore
AJ, 
Herd immunity likely protected the men who have sex with men in recent
hepatitis A outbreak in San Diego CA.
Clin Infect Dis
2018;25:25.10.1093/cid/ciy592PMC718212730052941299. Peak
CM, Stous
SS, Healy
JM,  Homelessness and hepatitis
A—San Diego County, 2016–2018. Clin Infect Dis 2019;ciz788.
10.1093/cid/ciz788PMC1095640231412358300. Wooten
DA. Forgotten but not gone:
learning from the hepatitis A outbreak and public health response in San
Diego.
Top Antivir Med
2019;26:117–21.30641485PMC6372360301. James
TL,
Aschkenasy
M, Eliseo
LJ, Olshaker
J, Mehta
SD. Response to hepatitis A
epidemic: emergency department collaboration with public health
commission.
J Emerg Med
2009;36:412–6.
10.1016/j.jemermed.2007.10.00118359602302. Moro
PL, Arana
J, Marquez
PL, 
Is there any harm in administering extra-doses of vaccine to a person?
Excess doses of vaccine reported to the Vaccine Adverse Event Reporting System
(VAERS), 2007–2017.
Vaccine
2019;37:3730–4.
10.1016/j.vaccine.2019.04.08831155414PMC6925972303. Iwarson
S, Lindh
M,
Widerström
L. Excellent booster response
4 to 8 years after a single primary dose of an inactivated hepatitis A
vaccine.
J Travel Med
2004;11:120–1.
10.2310/7060.2004.1707915109480304. Williams
JL, Bruden
DA, Cagle
HH, 
Hepatitis A vaccine: immunogenicity following administration of a delayed
immunization schedule in infants, children and adults.
Vaccine
2003;21:3208–11.
10.1016/S0264-410X(03)00250-012804849305. Hornick
R, Tucker
R, Kaplan
KM, 
A randomized study of a flexible booster dosing regimen of VAQTA in
adults: safety, tolerability, and immunogenicity.
Vaccine
2001;19:4727–31.
10.1016/S0264-410X(01)00224-911535323306. Schillie
S, Vellozzi
C, Reingold
A, 
Prevention of hepatitis B virus infection in the United States:
recommendations of the Advisory Committee on Immunization Practices.
MMWR Recomm Rep
2018;67(No. RR-1). 10.15585/mmwr.rr6701a129939980PMC5837403307. Bryan
JP, Henry
CH, Hoffman
AG, 
Randomized, cross-over, controlled comparison of two inactivated
hepatitis A vaccines.
Vaccine
2000;19:743–50.
10.1016/S0264-410X(00)00301-711115695308. Connor
BA, Phair
J, Sack
D, 
Randomized, double-blind study in healthy adults to assess the boosting
effect of Vaqta or Havrix after a single dose of Havrix.
Clin Infect Dis
2001;32:396–401.
10.1086/31852211170947309. CDC. FDA
approval of an alternate dosing schedule for a combined hepatitis A and B vaccine
(Twinrix®).
MMWR Morb Mortal Wkly Rep
2007;56:1057.310. Nothdurft
HD, Dietrich
M, Zuckerman
JN, 
A new accelerated vaccination schedule for rapid protection against
hepatitis A and B.
Vaccine
2002;20:1157–62.
10.1016/S0264-410X(01)00432-711803077

## Footnotes

* A third pediatric formulation of Havrix of 360 ELISA units per dose administered in a 3-dose schedule was recommended for persons aged 2–18 years in 1996 (*6**,**202*); however, by 1999 the pediatric formulation of 360 ELISA units per dose was no longer available and was no longer recommended (*7*).

## Appendix A Provider Guidance for Preexposure Protection for International Travelers Aged ≥12 Months

Hepatitis A (HepA) vaccination administered with the appropriate dose and schedule is preferred to immune globulin (IG) for preexposure protection for travelers aged ≥12 months. One dose of single-antigen HepA vaccine administered at any time before departure might provide adequate protection for most healthy persons (*1*). However, single-dose long-term protection data are limited, and limited data are available for other populations (e.g., immunocompromised) or other HepA vaccine formulations (i.e., Twinrix). For unvaccinated persons, the first dose of HepA vaccine should be administered as soon as travel is considered. The second dose should be administered according to the licensed schedule to complete the series. Travelers who have received a single dose of single-antigen HepA vaccine ≥6 months previously can receive a second dose of HepA vaccine to complete the vaccine series. Travelers who have received a single dose of single-antigen HepA vaccine within the previous 6 months do not need an additional dose for travel (*1*). Travelers may be administered Twinrix before travel, consisting of 3 doses administered on a 0-, 1-, and 6-month schedule. An alternate, accelerated schedule is also available for Twinrix: 3 doses at 0, 7, and 21–30 days, followed by a booster dose at 12 months that provides long-term protection (*2*).

For travelers aged >40 years, immunocompromised persons, and persons with chronic liver disease, the ability of the person to develop a protective antibody level after receipt of HepA vaccine, the magnitude of the risk for hepatitis A virus (HAV) transmission from the exposure (e.g., endemicity of hepatitis A in the area of travel), and availability of IG and vaccine should be considered in decisions to use IG in addition to vaccine (*1*). In addition, because of the complexity of determining HAV endemicity globally and limited data on subpopulation variation of HAV antibody (anti-HAV) seroprevalence within regions (*3*,*4*), preexposure protection against HAV infection can be considered for persons aged ≥12 months who are traveling to any area.

## Appendix B Provider Guidance on Risk Assessment for Hepatitis A Postexposure Prophylaxis

### Postexposure Prophylaxis for Persons Exposed to Hepatitis A Virus

Healthy persons who have completed the hepatitis A (HepA) vaccination series at any time do not need additional postexposure prophylaxis (PEP) if they are exposed to hepatitis A virus (HAV). The effectiveness of PEP is greater when administered soon after exposure. Therefore, if only immune globulin (IG) (*1*) or only vaccine is available for a person who has been exposed, either available product should be administered as soon as possible; the person may return for the other product if it becomes available within 2 weeks of exposure. If administered simultaneously, HepA vaccine and IG should be administered in a different anatomic site (i.e., separate limbs) (*2*). The HepA vaccine series should be completed with a second dose at least 6 months after the first dose for long-term protection.

PEP should be administered to all persons who have not previously completed the HepA vaccine series who have been exposed or are at risk for exposure through close personal contact with a person who has serologically confirmed HAV infection (e.g., household and sexual contacts, persons using injection or noninjection drugs with the HAV-infected person, or caregivers not using appropriate personal protective equipment) (*3*).

### Risk for HAV Transmission in Various Settings and for Various Groups

For persons exposed to HAV, considerations regarding decisions to use IG, vaccine, or both should include the ability of the person to develop a protective level of antibodies after receipt of HepA vaccine, the magnitude of the risk for HAV transmission from the exposure, and the availability of IG and vaccine (*4*). The severity of HAV infection and the time to develop protective antibodies increase with age (*4*). In general, the risk for infection after a household or sexual exposure is likely to be greater than the risk associated with a common source exposure (e.g., exposure to a contaminated food product or restaurant exposure) (*5*). The risk for transmission of HAV is influenced by host and environmental factors and varies considerably in different settings. For example, without PEP, secondary attack rates of 20%–50% have been reported in households, with higher rates of transmission occurring from infected young children than from infected adolescents and adults (*6*–*8*). In contrast, attack rates among patrons of food service establishments who have been exposed to HAV-infected food handlers are generally low (*5*,*9*–*12*). Given the difficulty in determining precise timing and extent of exposure and potential repeated exposures, as well as concerns over the administration and availability of IG (*1*,*11*), risk-based provider guidance is provided below.

**Child care centers.** PEP should be administered to all previously unvaccinated staff members and attendees of child care centers or institutions if 1) one or more cases of HAV infection are recognized in children or 2) cases are recognized in two or more households of center attendees. If one or more cases of HAV infection occur among employees, PEP for other staff and attendees should be considered based on the duties, hygienic practices, and presence of symptoms at work. In centers that do not provide care to children who wear diapers, PEP can be considered only for care center contacts of the index patient (*3*).

**Common-source food exposure and food handlers.** Because common-source transmission to patrons is unlikely (*5*,*9*,*11*,*12*), administering PEP to patrons of food establishments staffed by an HAV-infected person typically is not indicated. If, during the time when the food handler was likely to be infectious, the food handler both directly handled uncooked or cooked foods without gloves and had diarrhea or poor hygienic practices, the risk for an individual patron remains low; however, PEP may be considered for persons who have potentially been exposed (patrons or co-workers of the infected food handler during the time the food handler was symptomatic and worked) (*5*,*11*). PEP in this scenario should generally consist of vaccination for persons aged ≥12 months, although IG may be considered in addition to vaccine for exposed persons who are immunocompromised or have chronic liver disease. In settings in which repeated exposures to HAV might have occurred (e.g., institutional cafeterias), consideration of HepA vaccine, IG, or both is warranted.

**Settings providing services to children and adults.** PEP is not routinely indicated when a single case occurs in an elementary or secondary school or an office or other work setting and the source of infection is outside of the setting. PEP should be administered to persons who have close contact with index patients if an epidemiologic investigation indicates HAV transmission has occurred (e.g., among students in a school). PEP should be considered for all previously unvaccinated residents and employees when a confirmed hepatitis A case occurs in a setting where close personal contact occurs regularly and hygiene standards are difficult to maintain (e.g., correctional facility, homeless shelter, psychiatric facility, or group home or residential facility for persons with developmental disabilities). In a setting containing multiple enclosed units or sections (e.g., prison ward), PEP administration should be limited only to persons in the area with risk for exposure (*3*).

**Health care institutions.** Because health care personnel do not have an increased prevalence of HAV infection, HepA vaccination is not routinely recommended for health care personnel in the United States (*3*). When a person who has HAV infection is admitted to a hospital, staff members should not routinely be administered PEP; instead, appropriate infection control practices should be emphasized (i.e., contact precautions for diapered or incontinent patients) (*13*). PEP within the health care setting may be considered on a case-by-case basis if the risk for exposure to HAV is considered high. Outbreaks have been observed in neonatal intensive care units because of infants acquiring infection from transfused blood and subsequently transmitting HAV to other infants and staff (*14*,*15*). Outbreaks of hepatitis A caused by transmission from patients to health care personnel are typically associated with fecal incontinence and inadequate hand hygiene (*16*), although most hospitalized patients who have HAV infection are admitted after onset of jaundice, when they are beyond the point of peak infectivity (*17*,*18*). In hospitals, sharing food or beverages between patients, families, and health care personnel has been associated with HAV transmission (*19*,*20*).

Host-specific factors to consider when assessing for increased risk for HAV transmission in the health care setting include health care personnel with hepatitis A working during the infectious period or working with symptoms including diarrhea, and patients with hepatitis A who are diapered or incontinent and symptomatic, including diarrhea. Infection control considerations include poor compliance with hand hygiene, poor adherence to recommended standard and transmission-based precautions (e.g., contact precautions when caring for patients with hepatitis A who are diapered or incontinent, standard precautions for all others [https://www.cdc.gov/infectioncontrol/guidelines/isolation/index.html]) and suboptimal cleaning and disinfection of the environment. When perceived risk for HAV exposure is high, PEP for patients or health care personnel may be considered. In a health care setting with multiple enclosed units or sections (e.g., a hospital or psychiatric facility), PEP administration may be limited to areas where potential exposures may have occurred (e.g., cardiology ward or intensive care unit).
