# *Erratum:* Vol. 69, No. RR-5

**DOI:** 10.15585/mmwr.mm7008a5

**Published:** 2021-02-26

In the *MMWR Recommendations and Reports* “Prevention of Hepatitis A Virus Infection in the United States: Recommendations of the Advisory Committee on Immunization Practices, 2020,” an error occurred on page 11. In the third sentence of the second paragraph, the vaccines Havrix and Twinrix were incorrectly listed as having preservatives. This sentence should have read “**Havrix, Twinrix, and Vaqta are** formulated without a preservative.” 

